# Exploration of complex shyness and the relationship between fear and approach behaviors in domestic cats (*Felis catus*)

**DOI:** 10.1371/journal.pone.0360035

**Published:** 2026-11-04

**Authors:** Madoka Hattori, Atsuko Saito, Shinya Yamamoto

**Affiliations:** 1 Institute for the Future of Human Society, Kyoto University, Kyoto, Japan; 2 Department of Psychology, Faculty of Human Sciences, Sophia University, Tokyo, Japan; 3 Graduate School of Science, Kyoto University, Kyoto, Japan; Lyon College, UNITED STATES OF AMERICA

## Abstract

Shyness in human psychology is recognized as a complex trait distinct from pure fear, often characterized by an approach-avoidance conflict. While shyness and fear are frequently conflated in animal behavior studies, this research investigated shyness in domestic cats (*Felis catus*), exploring whether they exhibit multifaceted personality structures similar to those of human infants. We conducted three sequential surveys with cat owners: Study 1 included 82 cats of both sexes, Study 2 included 114 male cats to follow up on the sex-specific pattern observed in Study 1, and Study 3 included 114 stray-born male cats to further examine the pattern identified among stray-born cats in Study 2. We used adapted versions of the Colorado Childhood Temperament Inventory (CCTI) and the Infant Behavior Questionnaire-Revised (IBQ-R) to assess shyness, fear, and approach tendencies. The predicted quadratic relationship between shyness and approach scores was not statistically significant, and thus the primary confirmatory hypothesis was not supported. Subsequent exploratory hierarchical cluster analyses suggested four candidate personality phenotypes in stray-born male cats: “Highly Sociable” (low fear/high approach), “Sociable” (low fear/moderate approach), “Fearful” (high fear/low approach), and a “Human infant-like shyness” type (high fear/high approach). These findings suggest that cat shyness is predominantly linked to fear, as supported by the strong positive correlations between shyness and fear. However, the cluster analyses identified a specific subset of cats exhibiting a complex personality involving high-approach tendencies despite high fear, mirroring human infant-like shyness. This study highlights the multifaceted nature of cat temperament and demonstrates that adapting human psychological frameworks can provide significant insights into animal personality, potentially improving welfare in domestic cats.

## Introduction

Shyness is a complex personality trait in humans, and its characteristics and impacts have been extensively studied. Although shyness is similar to fear, studies suggest that shyness and fear are distinct personality traits in humans. Shyness is characterized by an excessive focus on oneself and undue concern for evaluations by others, leading to restrained behavior and a tendency to avoid social interactions [1]. Although both shyness and fear involve social anxiety and promote inhibition and avoidance, shyness is considered more complex than mere social anxiety [2]. For instance, studies on toddlers (18–30 months) showed that while shyness induces social tension and anxiety, fear is a reaction to direct threats [3]. Furthermore, experiments with human infants (7–13 months) showed that shyness in response to unfamiliar adults can be a conflicted state, characterized by a mix of desires to approach and also to withdraw due to fear, a condition known as “conflict” [4]. This study used the Colorado Childhood Temperament Inventory (CCTI) [5] rather than the five-factor model (FFM) [6], which includes five factors: openness to experience, conscientiousness, extraversion, agreeableness, and neuroticism. This is referred to as infant shyness, which is distinct from the fear-free self-conscious shyness observed from ages 4–5 years through adolescence [7].

However, in studies involving non-human animals, shyness and fear were not distinctly differentiated. For example, Charles Darwin described shyness as a form of fear, a defense mechanism evolving through the process of natural selection, and it is considered a common characteristic observed in both humans and other animals [8]. In personality investigations of mammals and fish, shyness was often used as an indicator of timidity, in contrast to being bold and active [9]. Similarly, in studies on the personality of dogs, the definition of shyness was used close to that of fear, alongside boldness [10]. In these studies, dog personality is initially parsed into five factors—’Playfulness,’ ‘Curiosity/Fearlessness,’ ‘Chase-proneness,’ ‘Sociability,’ and ‘Aggressiveness’—after which a higher order factor of shyness–boldness is employed. Thus, shyness is often positioned as the opposite of boldness and is assessed as a dimension of fear. This assumption has led to ignorance of the possible complexity of shyness in animals, as observed in humans.

Therefore, in this study, we investigated whether shyness as a complex personality trait (i.e., a mixture of fear and approach tendencies), as seen in humans, is observed in animals using the same approach as that used to evaluate shyness in human infants. Hence, we targeted cats (*Felis catus*) as subjects because they exhibit ambivalent behaviors such as varying levels of aggression toward their owners [11]. Cats have lived in association with humans for approximately 10,000 years and have been described as “semi-domesticated,” in part because their breeding has historically been subject to relatively limited human control [12]. Although few studies have directly compared the personality traits of dogs and cats, previous research has highlighted differences in their domestication histories and social behavior, with cats generally showing less social behavior than dogs [13]. Therefore, we considered cats as suitable animals for studying characteristics in which fear and approach are intermixed, similar to those of shyness described in human infants.

Previous studies of cat personality were not designed to separately assess shyness, fear, and approach, which were required to test the approach–avoidance hypothesis examined in this study. Although a variety of methods and scales have been developed for assessing personality traits of domestic cats, such as the Domestic Cat Personality Inventory [14], which analyzes six personality traits, the Feline Behavioral Assessment and Research Questionnaire (Fe-BARQ) [15], which conducts detailed behavioral analyses based on 100 questions, and the Feline-Ality™ assessment [16], which is designed to match shelter cats with potential owners. Although these have contributed to understanding cats’ personality, for example, by proposing a three-factor model comprising dominance, impulsiveness, and neuroticism [17], and a five-factor model comprising openness, quietness, affiliation, activity, and anthro-affiliation [18], the reliability and validity of these methods were not thoroughly verified, and no consensus exists on a unified approach [19]. More importantly, earlier studies have not analyzed complex personality traits such as shyness, the target trait in the current study.

Here, we focused on cats to explore shyness in non-human animals, hypothesizing that, similar to human infants, shyness in cats involves complex traits of fear and approach behaviors (hereafter, “complex shyness hypothesis”). Based on the human infant literature [4], we hypothesized that fear and approach tendencies may coexist in highly shy individuals. Because neither preverbal infants nor cats can verbally describe this conflict, it must be inferred from observable behavior or caregiver reports. For example, a cat with high fear and high approach tendencies may initially retreat from an unfamiliar person but continue to monitor the person and gradually move closer. This hypothesized pattern differs from that of a sociable cat that readily approaches with little avoidance and a fearful cat that remains withdrawn with little tendency to approach. In human infants, approach scores showed a quadratic relationship with shyness scores, with relatively high approach scores observed at both low and high levels of shyness [4]. We therefore examined whether the same quadratic pattern would be observed in cats. To test this hypothesis, we adapted the approach used in the previous study of human infants [4] to cats. We used a questionnaire designed to assess the shyness, personality, and behavioral characteristics of human infants. We selected the CCTI and IBQ-R because they separately assess shyness, fear, and approach, which were required to test the approach–avoidance hypothesis derived from the human infant literature. This adaptation was also based on a methodological parallel: in human infant research, an adult caregiver reports the behavior of a nonverbal infant, whereas in the present study, an adult owner reported the behavior of a nonverbal cat. In both cases, an adult familiar with the subject evaluates its observable behavior across everyday situations. The shyness score was derived from the CCTI [5], and the approach and fear scores were derived from the Infant Behavior Questionnaire-Revised (IBQ-R) [20,21]. The CCTI is a questionnaire that can measure shyness, and the IBQ-R is designed to measure fear and approach behaviors. We adapted these questionnaires for use with cats and their owners as exploratory operational measures, without assuming that the underlying constructs are equivalent in humans and cats.

## Study1

### Aims and hypotheses

If the complex shyness hypothesis were correct in cats, there would be a correlation between shyness and fear scores and a quadratic relationship between shyness and approach scores, as is observed in human infants [4]. Furthermore, we hypothesized that high approach scores could occur at both low and high shyness scores. This suggests that cats with moderate shyness scores might not exhibit approach, whereas those with extreme scores—either very low or very high—might still display approach behaviors.

### Methods

Before conducting the survey, pre-registration was completed (https://osf.io/b9v4y/).

### Ethics statement

The questionnaire surveys were approved by the research ethics committee and conducted under the guidelines for the ethics of research involving human participants established by the Wildlife Research Center of Kyoto University (approval number WRCH-2023–003). Before beginning the online questionnaire, all participants were provided with information about the study and provided electronic written informed consent by selecting the consent option.

### Participants

We recruited cat owners through social media between April 22 and 28, 2024. The cats included in this study were of any sex or breed. Based on the age classification proposed in an earlier study [22], the target population was aged 1–7 years old. All cats were spayed or neutered. Considering sex differences in relationships with cats [23], which suggest that women tend to have a stronger attachment to pets than do men, we specifically targeted women aged between 20 and 50 years. Survey responses were collected online, and no rewards were provided. The final sample size was 82 cats. The sample size was calculated based on a significance level (α) of 0.05, a power of 0.8, and an effect size (r) of 0.3. A total of 377 individuals expressed an interest in participating in the study. Random sampling was conducted to align with our sample size, and responses were collected from 82 cats (44 males and 38 females). The age of the cats ranged from 12 to 84 months (mean: 47.1 ±  SD 18.6, median: 47.0). The breed composition was predominantly mixed (n = 66), followed by Siberian (n = 5), British Shorthair (n = 3), Ragdoll (n = 3), Exotic Shorthair (n = 1), Somali (n = 1), Chinchilla Persian (n = 1), Norwegian Forest Cat (n = 1), and Maine Coon (n = 1) (S1 in S1 File). The age of the owners ranged from 20 to 50 years (mean: 38.1  ± SD 7.9, median: 39.5).

### Questionnaire

The questionnaire was originally designed for human infants and subsequently modified to align with cat characteristics. For example, the original item “Quickly becomes friendly with other children” was adapted to “Quickly becomes friendly with familiar people (non-family members with whom the cat has interacted several times) ”. The shyness score was derived from the Japanese version of the CCTI translated by Yoshitaka Matsuda [4,5]. The items were adapted to reflect cat behaviors. Before responding to these items, participants were instructed to evaluate their cats’ behavior toward humans. The items included the following statements: “My cat takes a long time to warm up to strangers,” “My cat tends to be shy,” “My cat makes friends easily” (reversed), “My cat is very friendly with strangers” (reversed), and “My cat is very sociable” (reversed). The response options ranged from 1 (not at all characteristic) to 5 (extremely characteristic).

The approach and fear scores were derived from the Japanese version of the IBQ-R translated by Atsuko Nakagawa [20,21,24]. The items were adapted to reflect the reactions of cats in various scenarios, such as “gets very excited when given a new toy,” “shows indifference when familiar relatives or friends of the owner visit,” “is startled by sudden or loud noises during daily activities,” and “seems anxious during the first few minutes of visiting a new place.” The response options ranged from 1 (never observed) to 7 (always observed), with the additional option X (not experienced, cannot answer). In the case of X, the item was deleted, and the individual average was calculated for the remaining items.

Cats display a range of responses to humans, with some exhibiting predominantly negative reactions compared to others. It is believed that the nature of cats that show negative reactions to humans differs from that of cats that do not. To examine the specific response of cats to their owners, we added the following three items of negative behaviors: “hesitates to approach the owner,” “resists being picked up by the owner,” and “runs away or hides when the owner approaches.” The response options ranged from 1 (never observed) to 7 (always observed), with the additional option X (not experienced, cannot answer). In the case of X, the item was deleted, and the individual average was calculated for the remaining items. Additionally, data pertaining to the age of the owner, age of the cat, and number of years with the cat were collected.

The variables included shyness (on a scale of 1–5), approach (on a scale of 1–7), fear (on a scale of 1–7), and owner interaction (on a scale of 1–7). The shyness score comprised 5 items, and the total score is calculated by summing these items (minimum and maximum scores of 5 and 25, respectively). The approach, fear, and interaction scores consisted of 12, 16, and 3 items, respectively. Each of these was calculated as an average on a scale of 1–7 (minimum and maximum scores of 1 and 7, respectively).

### Statistical analysis

Spearman’s rank correlation was used to examine the correlation coefficients between the shyness score and fear scores, considering the skewness of the data. A linear regression model with a quadratic term was used to analyze the relationship between the shyness score and the approach score. The quadratic term was included based on our hypothesis that the relationship between shyness and approach would be nonlinear, with approach tendencies differing across low, intermediate, and high levels of shyness rather than following a simple linear pattern. The analysis was performed using the R function lm (Approach ~ Shyness + I(Shyness^2)). An exploratory analysis of the three newly added items regarding the interaction of the cat with its owner was conducted using Spearman’s rank correlation to assess the correlation between these factors and the shyness score. This study aimed to evaluate the interaction of the cats with their owners. To verify the internal consistency of the questionnaire, Cronbach’s alpha was calculated. A previous study reported sex differences in several behavioral traits in cats, including nervousness [25]. Therefore, we conducted exploratory correlational analyses separately for male and female cats. All analyses were conducted using R statistical software version 4.2.2.

### Results

Cronbach’s alpha demonstrated high reliability for the measured scales, with the shyness, fear, and approach scores at α = 0.95, 0.88, and 0.81, respectively. Spearman’s rank correlation showed a strong positive correlation between shyness and fear scores (*rs* = 0.788, *p* < 0.001, n = 82). The relationship between the approach and the shyness score is shown in Table 1. The quadratic term had no significant effect (*p* = 0.170). A tendency toward a negative correlation was observed in the linear term (*p* = 0.052). The correlation between fear scores and approach scores was weakly negative (*rs* = − 0.305, *p* = 0.005).

**Table 1:** The results of the quadratic relationship analysis of cats (n = 82). The final line summarizes the model’s fit and statistical significance.

Variable	Estimate	*SE*	*β*	*t-*value	*p-*value	95% CI
Intercept	6.432	0.661		9.735	< 0.001	5.116 - 7.747
Shyness	−0.192	0.098	−1.208	−1.971	0.052	−0.386 - 0.002
Shyness^2	0.004	0.003	0.849	1.385	0.170	−0.002 - 0.011

*R*² = 0.158, Adjusted *R*² = 0.137, *F*-ratio = 7.43, *p* = 0.001.

Exploratory analyses indicated no correlation between shyness scores and negative behaviors toward owners (*rs* = 0.063, *p* = 0.347). We additionally conducted exploratory analyses separately for male and female cats. In female cats (n = 38), a positive correlation existed between the shyness and fear scores (*rs* = 0.861, *p* < 0.001). The quadratic relationship between shyness and approach scores was not significant (quadratic term p = 0.202) (Table 2). In male cats (n = 44), a positive correlation existed between shyness and fear scores (*rs* = 0.713, *p* < 0.001). The quadratic relationship between shyness and approach scores was also not statistically significant (quadratic term *p* = 0.131) (Table 3, Fig 1 and 2).

**Table 2:** The results of the quadratic relationship analysis of female cats (n = 38). The final line summarizes the model’s fit and statistical significance.

Variable	Estimate	*SE*	*β*	*t*-value	*p*-value	95% CI
Intercept	7.089	1.105		6.414	<0.001	4.845 - 9.333
Shyness	−0.290	0.157	−1.654	−1.842	0.074	−0.610 - 0.030
Shyness^2	0.006	0.005	1.168	1.301	0.202	−0.004 - 0.016

*R*² = 0.285, Adjusted *R*² = 0.244, *F*-ratio = 6.982, *p* = 0.003.

**Table 3:** The results of quadratic relationship analysis of male cats (n = 44). The final line summarizes the model’s fit and statistical significance.

Variable	Estimate	*SE*	*β*	*t*-value	*p*-value	95% CI
Intercept	6.304	0.719		8.774	< 0.001	4.853 - 7.755
Shyness	−0.187	0.110	−1.453	−1.710	0.095	−0.409 - 0.034
Shyness^2	0.006	0.004	1.311	1.543	0.131	−0.002 - 0.013

*R*² = 0.080, Adjusted *R*² = 0.035, *F*-ratio = 1.779, *p* = 0.181.

**Fig 1:**
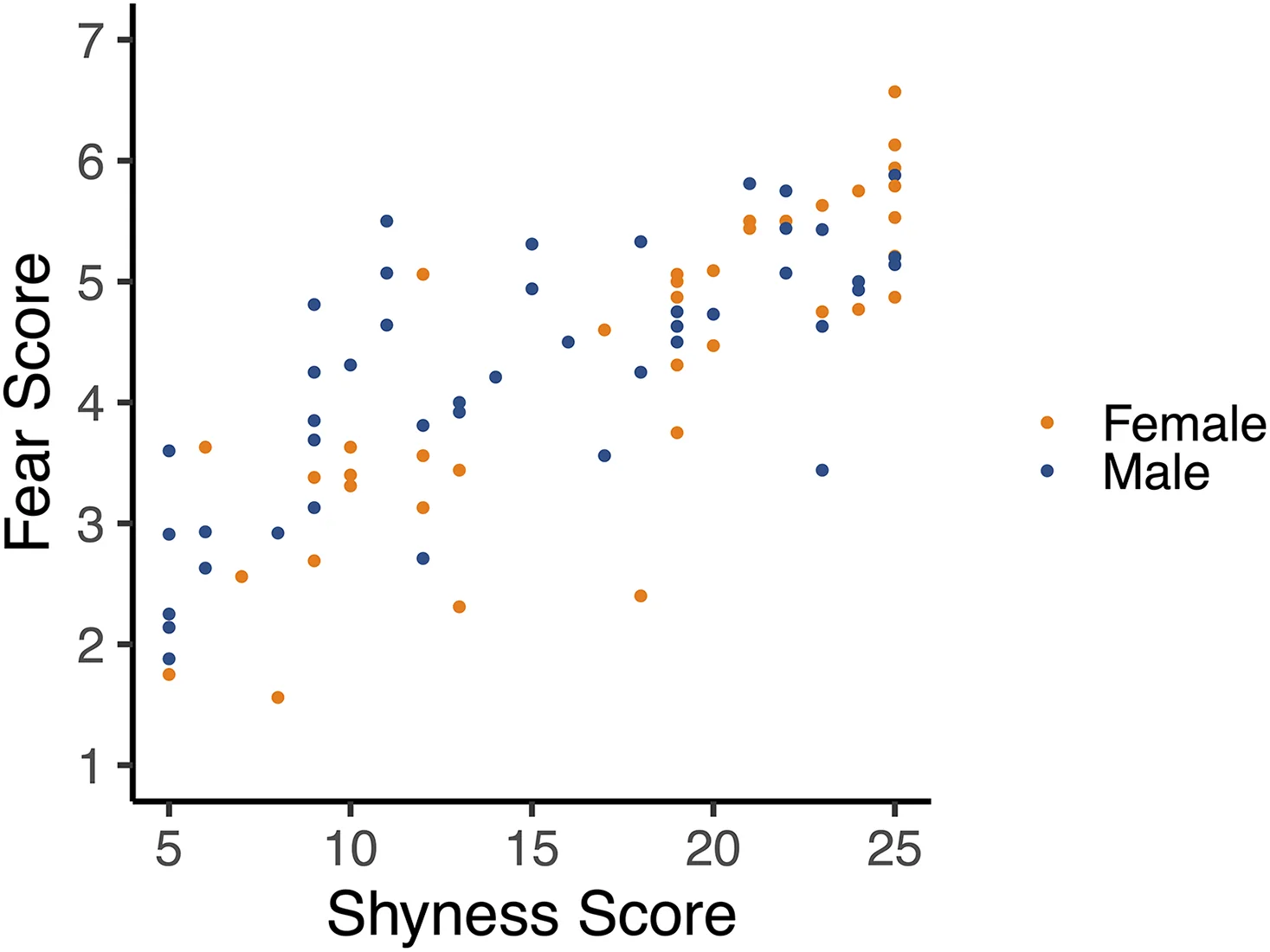
Scatter plot of shyness scores and fear scores, divided by sex. Females (n = 38) and males (n = 44).

**Fig 2:**
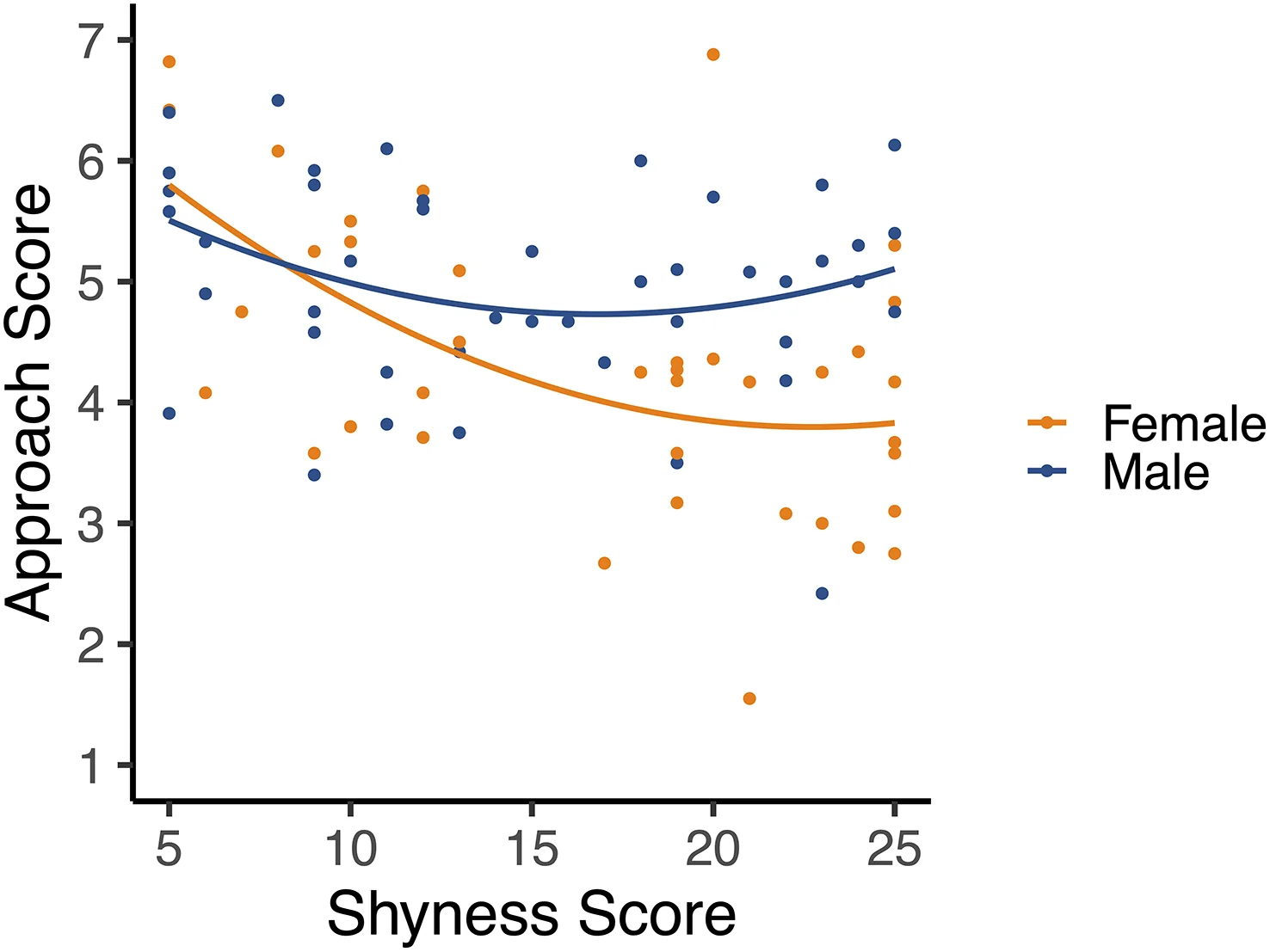
Scatter plot of shyness scores and approach scores, divided by gender. Females (n = 38) and males (n = 44). The solid lines represent quadratic regression curves based on the model that includes both a linear and a squared term for shyness for each sex.

A post-hoc power analysis was conducted to verify the statistical validity of the results between shyness and approach scores. For all cats (n = 82), the effect size of Cohen’s f^2^ = 0.188 with α = 0.05 resulted in a power of 0.941. For female cats (n = 38), the effect size of Cohen’s f² = 0.399 with α = 0.05 resulted in a power of 0.926. In the case of male cats (n = 44), the effect size of Cohen’s f² = 0.087 with α = 0.05 resulted in a power of 0.374.

### Discussion

As predicted, a positive correlation was observed between shyness and fear. However, contrary to our prediction, no quadratic relationship was observed between shyness and approach scores. Therefore, these results do not support the complex shyness hypothesis. However, further exploratory analyses revealed differences in the relationship between shyness and approach scores between male and female cats. The quadratic relationship may be weak or absent in male cats; however, the possibility that it was not detected because of the small number of male cats in Study 1 could not be excluded. Therefore, we conducted Study 2 with an expanded sample of male cats to further examine the quadratic relationship.

## Study2

### Aims and hypotheses

Based on the findings of Study 1, a new hypothesis, specifically focusing on male cats, was formulated. We hypothesized that shyness in male cats, similar to that in human infants, is a combination of fear and approach traits. If this hypothesis were correct, a positive correlation would be observed between shyness and fear scores, whereas a quadratic relationship would be observed between shyness and approach scores. Thus, this is a replication of Study 1, although it focused on male cats and increased the sample size to obtain sufficient statistical power.

### Methods

Pre-registration was completed before the survey. (https://osf.io/zns5m/)

The ethical approval and informed consent procedures were the same as those described in Study 1.

### Participants

Based on the results obtained from the male cat cohort in Study 1, the sample size was set to 114. This number was calculated with a significance level of 0.05, power of 0.8, and Cohen’s f^2^ effect size of 0.087. The study population consisted exclusively of neutered male cats aged 1–7 years, irrespective of breed. Participants were limited to women between the ages of 20 and 50. Totally, 144 data points were collected between May 14 and 15, 2024. To align with the targeted sample size of n = 114, participants whose responses indicated “did not answer due to lack of experience” over 50% were excluded (n = 6), followed by random selection from the remaining respondents. The age of the cats ranged from 12–84 months (mean: 51.8 ± SD 20.2, median: 55.0), and all were neutered. The breed composition included mostly mixed-breed cats (n = 93), with smaller numbers of American Shorthair (n = 3), Exotic Shorthair (n = 3), Siberian (n = 2), Chartreux (n = 2), Somali (n = 2), Norwegian Forest Cat (n = 2), and Maine Coon (n = 2), and one each of Abyssinian, American Curl, Bengal, Ragamuffin, and Munchkin (S2 in S1 File). The age of the owners ranged from 20 to 50 (mean: 37.2 ± SD 6.7, median: 35.5).

### Questionnaire

Data were collected using the same questionnaire and variables utilized in Study 1.

### Statistical analysis

Spearman’s rank correlation was used to examine the correlation coefficients between the shyness and approach and fear scores. A linear regression model with a quadratic term was used to analyze the relationship between the shyness score and the approach score. The analysis was performed using the R function lm (Approach ~ Shyness + I(Shyness^2)). Furthermore, an exploratory cluster analysis based on shyness, fear, and approach scores was conducted to identify groups characterized by different combinations of these traits. In particular, we examined whether a subgroup with both high fear and high approach tendencies, as predicted by the complex shyness hypothesis, could be identified. As additional exploratory analyses not specified in the preregistration, we examined differences in cat age and childhood background among the clusters. Cat age was compared using a Kruskal–Wallis test, and childhood background was compared using Fisher’s exact test. The analyses were conducted using R statistical software, version 4.2.2.

### Results

Spearman’s rank correlation analysis indicated a significant positive correlation between shyness and fear scores (*rs* = 0.846, p < 0.001). No quadratic relationship was observed between shyness and approach scores, and this correlation was not statistically significant (quadratic term *p* = 0.269) (Table 4). Consequently, a post-hoc power analysis was conducted, resulting in an effect size of Cohen’s f^2^ = 0.289, with a sample size of n = 114 and α = 0.05, which yielded a power of 1. The correlation between fear scores and approach scores was weak (*rs* = 0.324, *p* < 0.001).

**Table 4:** The results of quadratic relationship analysis of male cats (n = 114). The final line summarizes the model’s fit and statistical significance.

Variable	Estimate	*SE*	*β*	*t*-value	*p*-value	95% CI
Intercept	6.082	0.446		13.623	< 0.001	5.197 - 6.966
Shyness	−0.140	0.073	−1.084	−1.922	0.057	−0.284 - 0.004
Shyness^2	0.003	0.002	0.627	1.112	0.269	−0.002 - 0.007

*R*² = 0.224, Adjusted *R*2 = 0.210, *F*-ratio = 16.02, *p* < 0.001.

A cluster analysis was conducted as part of the exploratory analysis. The initial step involved scaling (standardizing) the variables of interest, namely, shyness, approach, and fear scores. Subsequently, hierarchical clustering was performed on the standardized data using Ward’s method. The resulting dendrograms were used to visualize the clustering outcomes, and the optimal number of clusters was determined to be k = 4 based on the elbow method (Fig 3, Fig 4, Fig 5, and 6). Cluster 1 consisted of 21 cats, with shyness, fear, and approach average scores of 6.143, 2.593, and 5.929, respectively. Cluster 2 had 33 participants, with shyness, fear, and approach scores of 10.061, 3.240, and 4.618, respectively. Cluster 3 had 48 participants, with shyness, fear, and approach average scores of 21.813, 5.285, and 4.656, respectively. Cluster 4 consisted of 12 participants, with shyness, fear, and approach scores of 23.750, 5.263, and 2.875, respectively (Table 5).

**Table 5:** Mean shyness, fear, and approach scores by cluster (with standard deviations in parentheses) in male cats (n = 114).

Cluster	Numbers	Shyness Scores (5–25)	Fear Scores (1–7)	Approach Scores (1–7)
1	n = 21	6.143 (1.389)	2.593 (0.799)	5.929 (0.472)
2	n = 33	10.061 (4.107)	3.240 (0.772)	4.618 (0.735)
3	n = 48	21.813 (3.630)	5.285 (0.588)	4.656 (0.550)
4	n = 12	23.750 (1.357)	5.263 (0.507)	2.875 (0.610)

**Fig 3:**
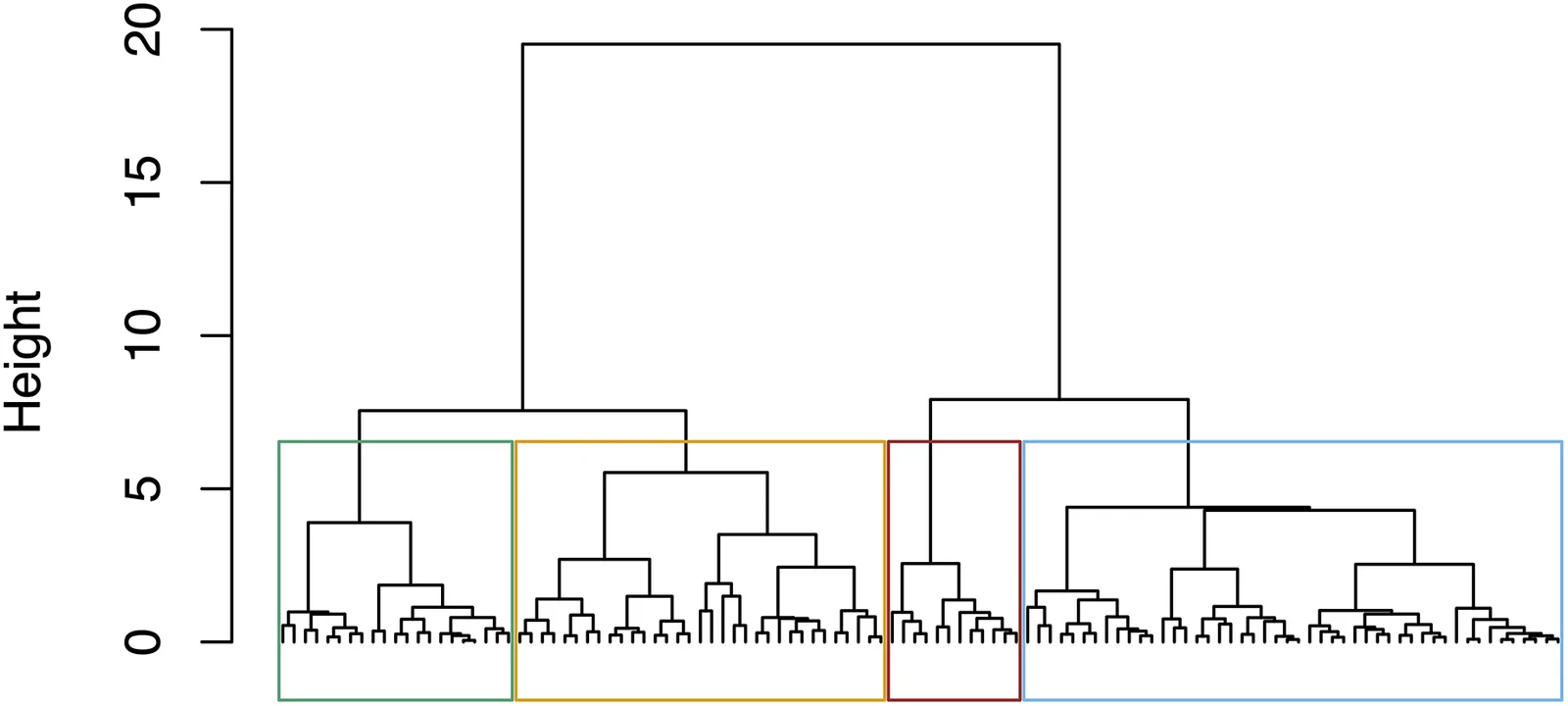
Dendrogram of male cats. Results of the cluster analysis indicated that the personality traits of male cats can be divided into four distinct clusters.

**Fig 4:**
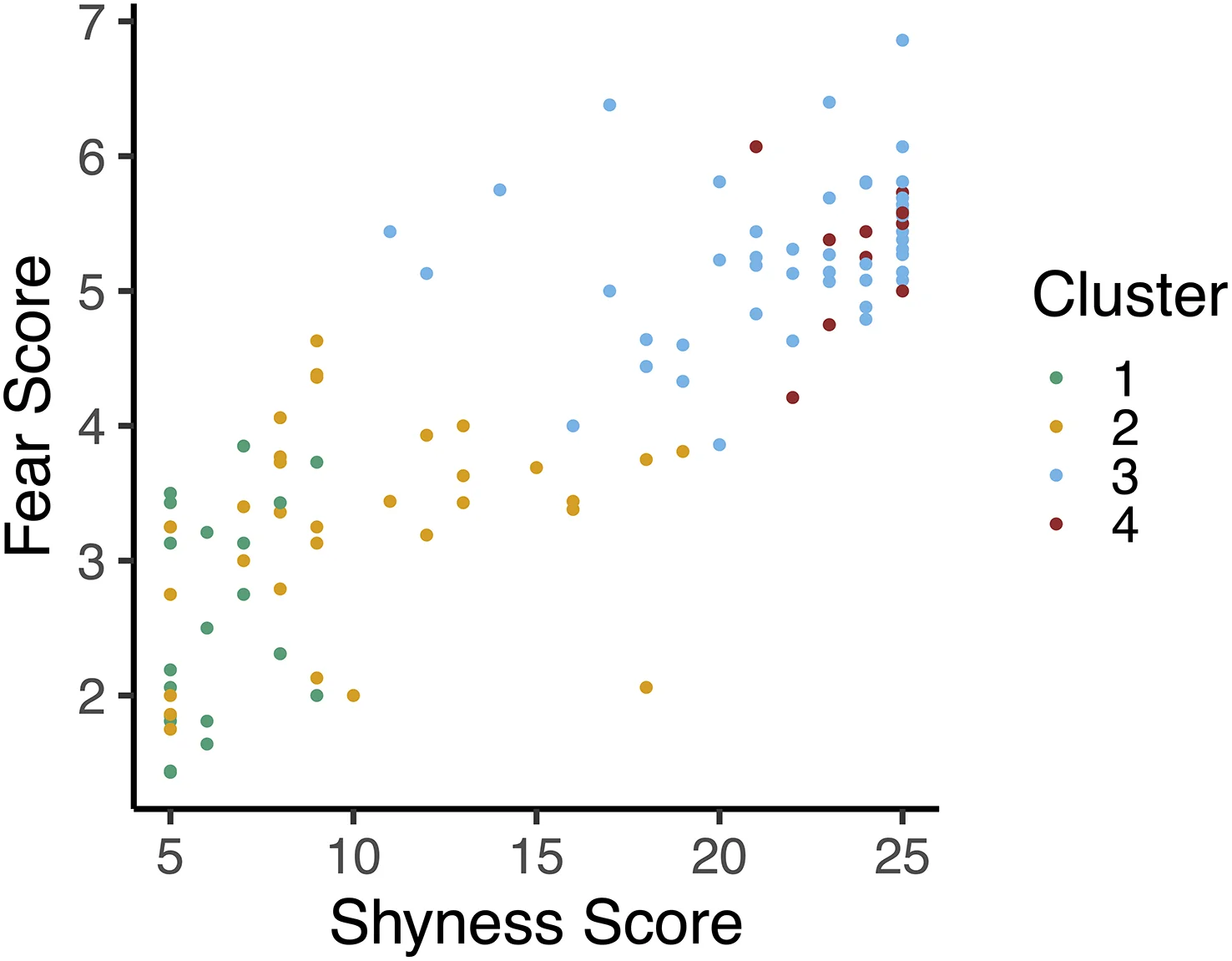
Scatter plot of shyness and fear scores, color-coded by cluster in male cats (n = 114).

**Fig 5:**
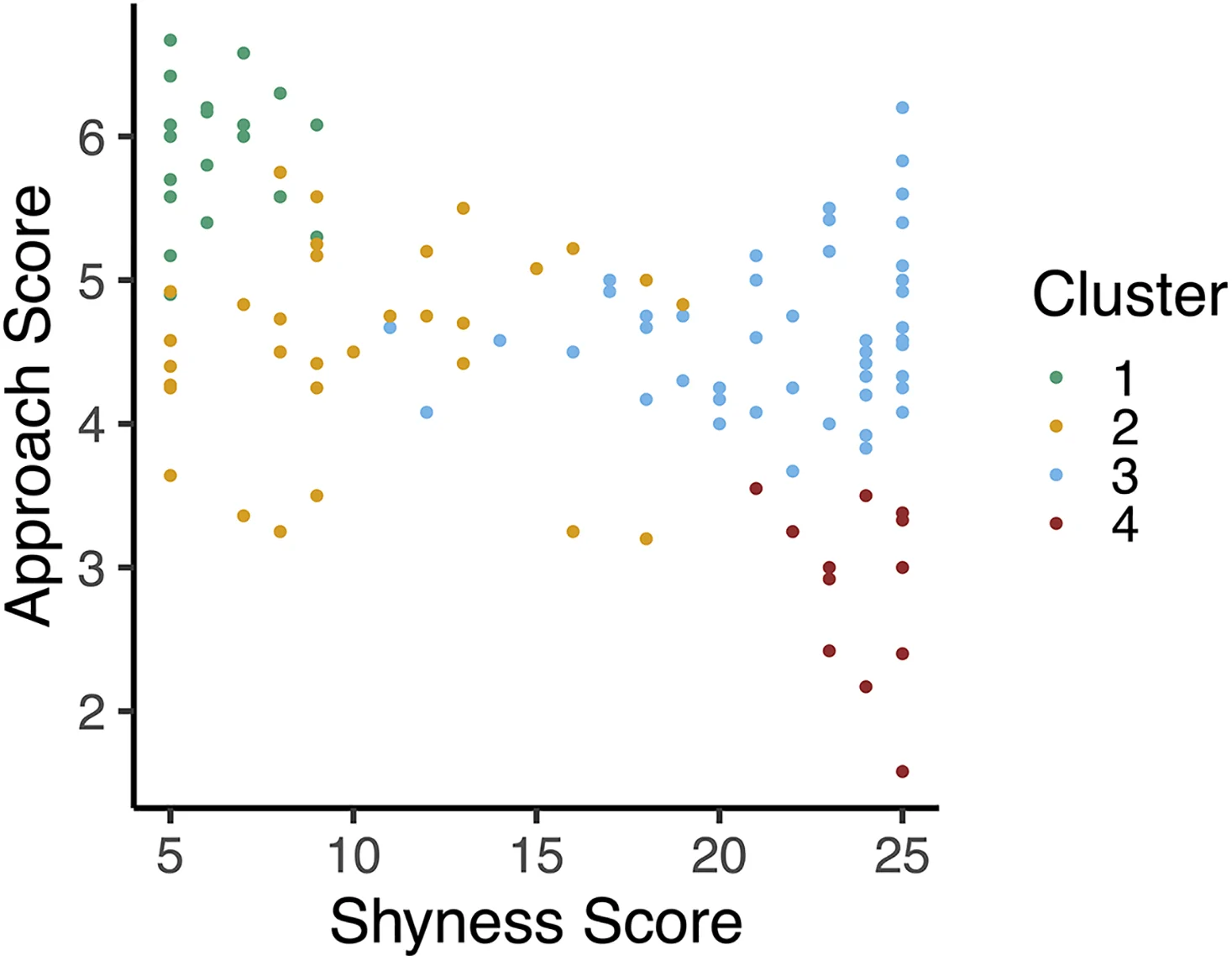
Scatter plot of shyness and approach scores, color-coded by cluster in male cats (n = 114).

**Fig 6:**
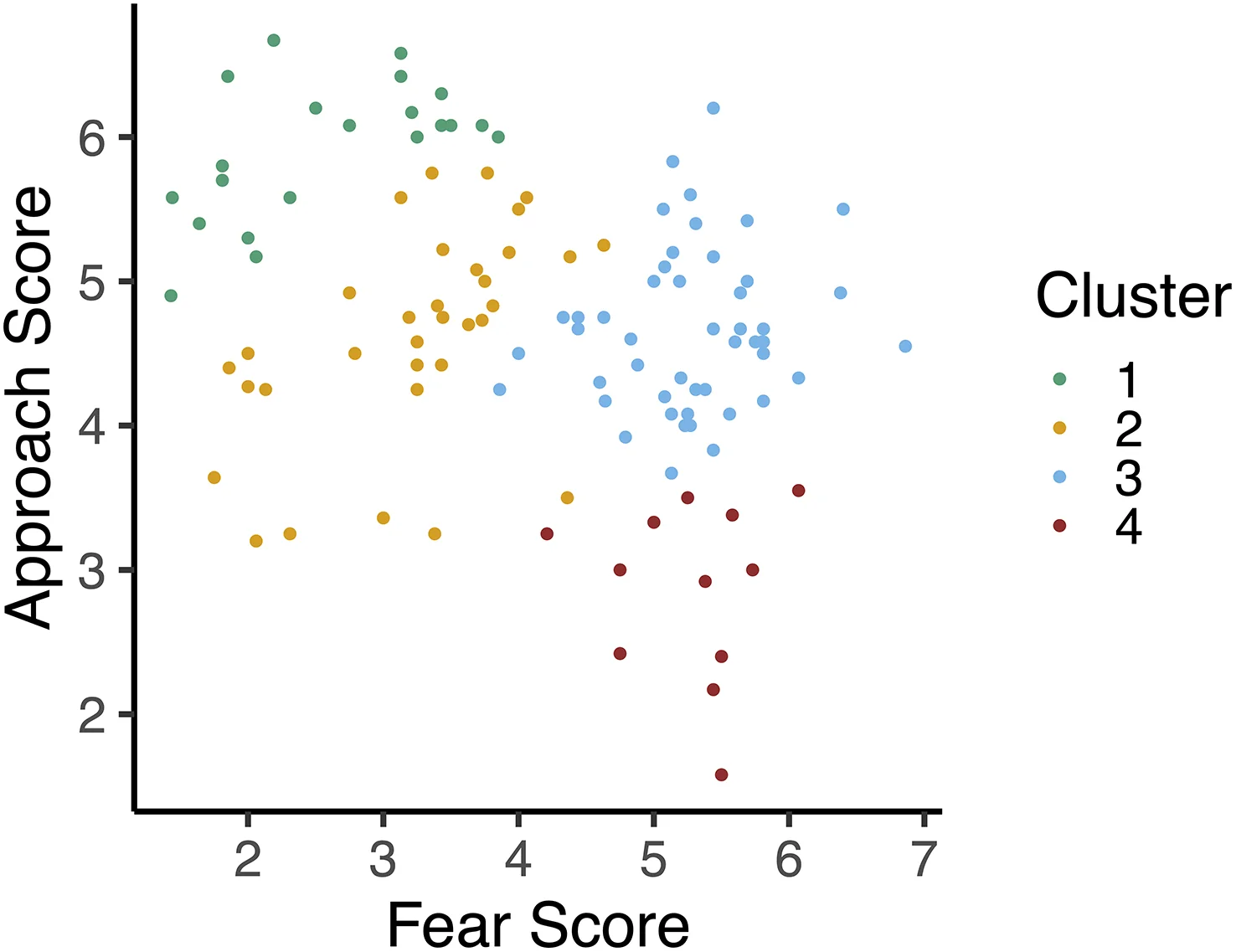
Scatter plot of fear and approach scores, color-coded by cluster in males (n = 114).

Furthermore, the relationship between cat backgrounds and their cluster groupings was analyzed. The results showed that in Cluster 1, purebred cats in contact with humans since birth were n = 5, cats born stray were n = 6, and cats with unknown backgrounds were n = 10. In Cluster 2, purebred cats in contact with humans since birth were n = 9, cats born stray were n = 13, and cats with unknown backgrounds were n = 11. In Cluster 3, purebred cats in contact with humans since birth were n = 4, cats born stray were n = 23, and cats with unknown backgrounds were n = 21. In Cluster 4, purebred cats in contact with humans since birth were n = 2, cats born stray were n = 7, and cats with unknown backgrounds were n = 3 (Table 6). The distribution of childhood background did not significantly differ among the four clusters (Fisher’s exact test, *p* = 0.201).

**Table 6:** Background of male cats (n = 114) by cluster.

Cluster	Numbers	Purebred cat (n = 20)	Stray cat (n = 49)	Unclear (n = 45)
1	n = 21	5 (25%)	6 (12.2%)	10 (22.2%)
2	n = 33	9 (45%)	13 (26.5%)	11 (24.4%)
3	n = 48	4 (20%)	23 (46.9%)	21 (46.7%)
4	n = 12	2 (10%)	7 (14.3%)	3 (6.7%)

The relationship between the age of cats in months and their cluster groupings is as follows: Clusters 1, 2, 3, and 4 had a mean of 50.2 ± SD 21.4, median 59; mean of 51.8 ± SD 21.2, median 54; mean of 51.0 ± SD 19.8, median 50.5; and mean of 57.8 ± SD 18.0, median 59.5, respectively (Table 7). The age of cats did not significantly differ among the four clusters (Kruskal–Wallis test, χ^2^(3) = 1.54, *p* = 0.673).

**Table 7:** Age in months of male cats (n = 114) by cluster.

Cluster	Numbers	Mean	SD	Median
1	n = 21	50.2	21.4	59
2	n = 33	51.8	21.2	54
3	n = 48	51.0	19.8	50.5
4	n = 12	57.8	18.0	59.5

The distribution of birth backgrounds across the four clusters is shown in Table 6. For stray cats (n = 49), the relationship between age in months and cluster groupings is as follows: Cluster 1, 2, 3, and 4 had a mean of 52.2 ± SD 17.4, median 59.5; mean of 55.7 ± SD 18.4, median 60; mean of 50.9 ± SD 21.2, median 51; and mean of 62.7 ± SD 18.6, median 60, respectively (Table 8). Age and month of breeding were highly correlated (*r* = 0.95).

**Table 8:** Age in months of stray male cats (n = 49).

Cluster	Numbers	Mean	SD	Median
1	n = 6	52.2	17.4	59.5
2	n = 13	55.7	18.4	60
3	n = 23	50.9	21.2	51
4	n = 7	62.7	18.6	60

### Discussion

A strong positive correlation was identified between shyness and fear scores in male cats, whereas no quadratic relationship was observed for the approach score. These results did not support the hypothesis of complex shyness in male cats.

However, further exploratory analysis, specifically a cluster analysis using the shyness score and both the approach and fear scores, revealed that male cats can be classified into four distinct clusters based on their traits: Cluster 1 is characterized by the lowest scores for shyness and fear, accompanied by a high approach score, suggesting a “Highly Sociable” type; Cluster 2 exhibits low levels of shyness and fear, yet displays a high approach score, thus categorizing it as a “Sociable” type; Cluster 3 exhibits elevated levels of shyness and fear, accompanied by a high approach score, which suggests a “human infant-like shyness” type, wherein fear and approach traits are intertwined to form human infant-like shyness traits; Cluster 4 is distinguished by elevated levels of shyness and fear, accompanied by a diminished approach score, which categorizes it as a “Fearful” type (Table 5). This may align with human infants, as their traits can be classified based on a combination of social approach and avoidance behaviors that vary depending on the level of each, creating distinct behavioral types [26,27]. For example, individuals exhibiting both high approach and avoidance behaviors are classified as “conflicted shy,” whereas those displaying low approach and high avoidance are labeled “avoidant.” Those with low approach and avoidance are referred to as “introverts,” and those with high approach and low avoidance are known as “sociable.” The results of the current study suggest that the personality traits of cats can be divided into several categories.

Furthermore, our data showed that stray cats accounted for 46.9% of Cluster 3 (human infant-like shyness type) (Table 6). Additionally, it was determined that among the cats born stray (n = 49), those classified as the human infant-like shyness type tended to be younger than the remaining subjects (Table 8). Although neither childhood background nor age differed significantly among the four clusters, these descriptive patterns raise the possibility that some cats are initially born stray with traits of human infant-like shyness, which may subsequently transition into sociable or fearful types as they age or live longer with humans. As this study focused on cats younger than seven years, the influence of aging was considered to be less significant than that of the impact of human interaction. Accordingly, Study 3 was conducted to investigate whether the cluster types changed because of human interactions.

## Study3

### Aims and hypotheses

Based on the results of Study 2, we developed new follow-up hypotheses regarding natal origins and developmental changes in Human infant-like shyness. Study 2 focused on male cats because it followed up on the exploratory finding in Study 1 that shyness was positively associated with approach in male cats but not in female cats. Therefore, the hypotheses derived from Study 2 were specific to male cats. Furthermore, Study 2 identified the Human infant-like shyness type, characterized by high fear and high approach, among stray-born male cats. We therefore focused on this natal background in Study 3 and hypothesized that stray-born male cats would be more likely to display this personality type (Hypothesis 1). We further developed hypotheses on developmental changes after interacting with human caregivers in human environments. This can be categorized into two sub-hypotheses: One (Hypothesis 2–1) posits a transition to a more sociable type, characterized by a reduction in fear behaviors and the maintenance of high-approach behaviors. This leads to the prediction that the months of breeding a cat has been bred will demonstrate a negative correlation with fear scores, whereas no correlation will be observed with approach scores, which typically remain high. The other sub-hypothesis (Hypothesis 2–2) posits that stray male cats become either sociable, exhibiting fewer fear behaviors and high approach behaviors, or fearful, displaying high fear and low approach behaviors. If this hypothesis is correct, either a negative correlation between the months of breeding and the fear score, but no correlation with the approach score, or no correlation between the months of breeding and the fear score, but a negative correlation with the approach score, will be exhibited. To test these hypotheses, a new study was conducted, focusing exclusively on male cats originally stray, with sufficient statistical power and an increased sample size of stray cats.

### Methods

Pre-registration was completed prior to the survey (https://osf.io/g28xd/).

The ethical approval and informed consent procedures were the same as those described in Study 1.

### Participants

As preregistered, the target sample size for Study 3 was set at 114, following the sample size used in Study 2. Because Study 3 involved a different hypothesis and statistical analysis, this sample size was not based on a study-specific power analysis. This study focused solely on neutered male cats aged 1–7 years, specifically those born as strays. Women between the ages of 20 and 50 years were selected. Data were collected between July 13 and 16, 2024. In total, 264 data points were collected. To align with the desired sample size of n = 114, cats with unknown backgrounds were excluded (n = 74), followed by the exclusion of those surveys where responses to “did not answer due to lack of experience” exceeded 50% (n = 9), after which random sampling was conducted. The cats’ ages ranged from 12 to 84 months (47.6 ±  SD 21.7, median: 47.0), and all were neutered. The cats’ months of breeding ranged from 6 to 82 months (41.2 ±  SD 22, median: 40). The age of the owners ranged from 20 to 50 years (mean: 37.4  ±  SD 6.9, median: 36.5) (S3 in S1 File).

### Questionnaire

Data were collected using the same questionnaire and variables utilized in Study 1.

### Statistical analysis

To control for the effect of age, Spearman’s rank correlation was calculated between shyness, fear, and approach scores and months of breeding. Additionally, as part of the exploratory analysis, cluster analysis was performed using Ward’s method, similar to that employed in Study 2. As additional exploratory analyses not specified in the preregistration, cat age and months of breeding were compared among the clusters using Kruskal–Wallis tests. When a significant difference was detected, post hoc pairwise Wilcoxon rank-sum tests with Holm correction were performed. Analyses were conducted using the R statistical computing platform, version 4.2.2.

### Results

The results of the partial correlation analysis, controlling for the effect of age, indicated no significant correlation between shyness (*rs* = 0.002, *p* = 0.986), fear (*rs* = 0.014, *p* = 0.886), and approach (*rs* = 0.150, *p* = 0.113) scores, respectively, and the number of breeding months. Visual analysis of the scatter plots for shyness (Fig 7), fear (Fig 8), and approach (Fig 9) scores and months of breeding, showed no evidence of the predicted divergence into the two groups, as postulated in hypothesis 2–2. Further exploratory analysis included a cluster analysis, which revealed that personality traits could be divided into four clusters (Fig 10). Cluster 1 consisted of 15 participants, with shyness, fear, and approach scores of 7.400, 2.503, and 6.223, respectively. Cluster 2 consisted of 41 participants, with shyness, fear, and approach scores of 9.561, 3.324, and 4.648, respectively. Cluster 3 had n = 35, with shyness, fear, and approach scores of 22.400, 5.235, and 4.914, respectively. Cluster 4 had 23 participants, with shyness, fear, and approach scores of 21.522, 5.396, and 3.530, respectively (Table 9). The average number of months of breeding for each cluster was as follows: Cluster 1 was mean: 27.4 ±  SD20.3, median: 22, Cluster 2 was mean: 43.7 ±  SD21.8, median: 45, Cluster 3 was mean: 42.1 ±  SD21.7, median: 41, and Cluster 4 was mean: 44.4 ±  SD21.8, median: 48 (Table 10). The number of months of breeding did not significantly differ among the four clusters (Kruskal–Wallis test, χ^2^(3) = 7.35, *p* = 0.062). Cat age differed significantly among the four clusters (Kruskal–Wallis test, χ^2^(3) = 8.25, *p* = 0.041); however, post hoc pairwise comparisons with Holm correction showed no significant differences between individual clusters (all adjusted *p* > 0.05).

**Table 9:** Mean shyness, fear, and approach scores by cluster (with standard deviations in parentheses).

Cluster	Numbers	Shyness Scores (5–25)	Fear Scores (1–7)	Approach Scores (1–7)
1	n = 15	7.400 (2.780)	2.503 (0.972)	6.223 (0.422)
2	n = 41	9.561 (3.139)	3.324 (0.939)	4.648 (0.651)
3	n = 35	22.400 (2.172)	5.235 (0.665)	4.914 (0.433)
4	n = 23	21.522 (2.660)	5.396 (0.464)	3.530 (0.627)

**Table 10:** Months of breeding in months of cats by cluster.

Cluster	Numbers	Mean	SD	Median
1	n = 15	27.4	20.3	22
2	n = 41	43.7	21.8	45
3	n = 35	42.1	21.7	41
4	n = 23	44.4	21.8	48

**Fig 7:**
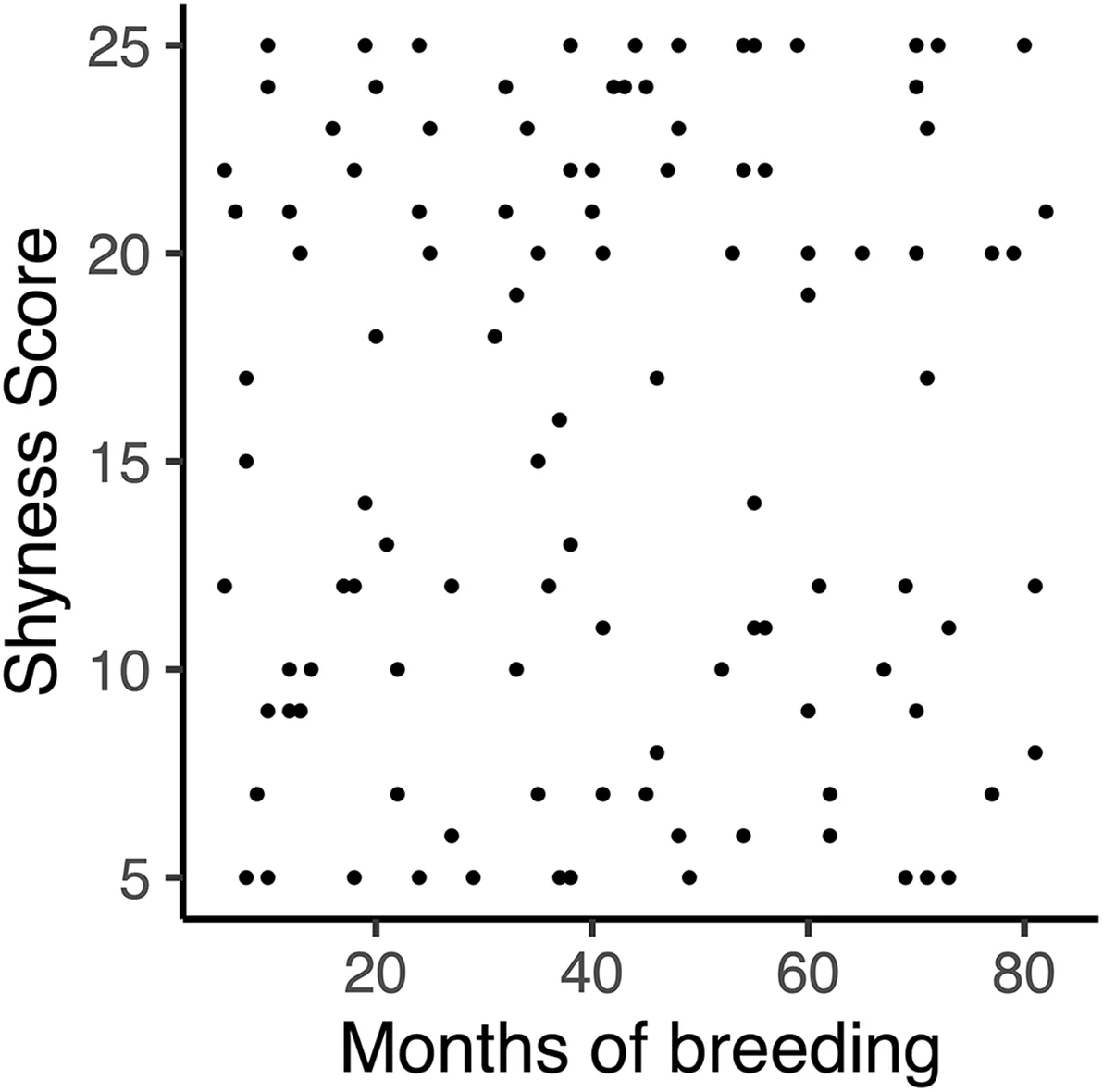
Scatter plot of months of breeding and shyness scores (r = 0.063, p = 0.507) in males born as strays (n = 114).

**Fig 8:**
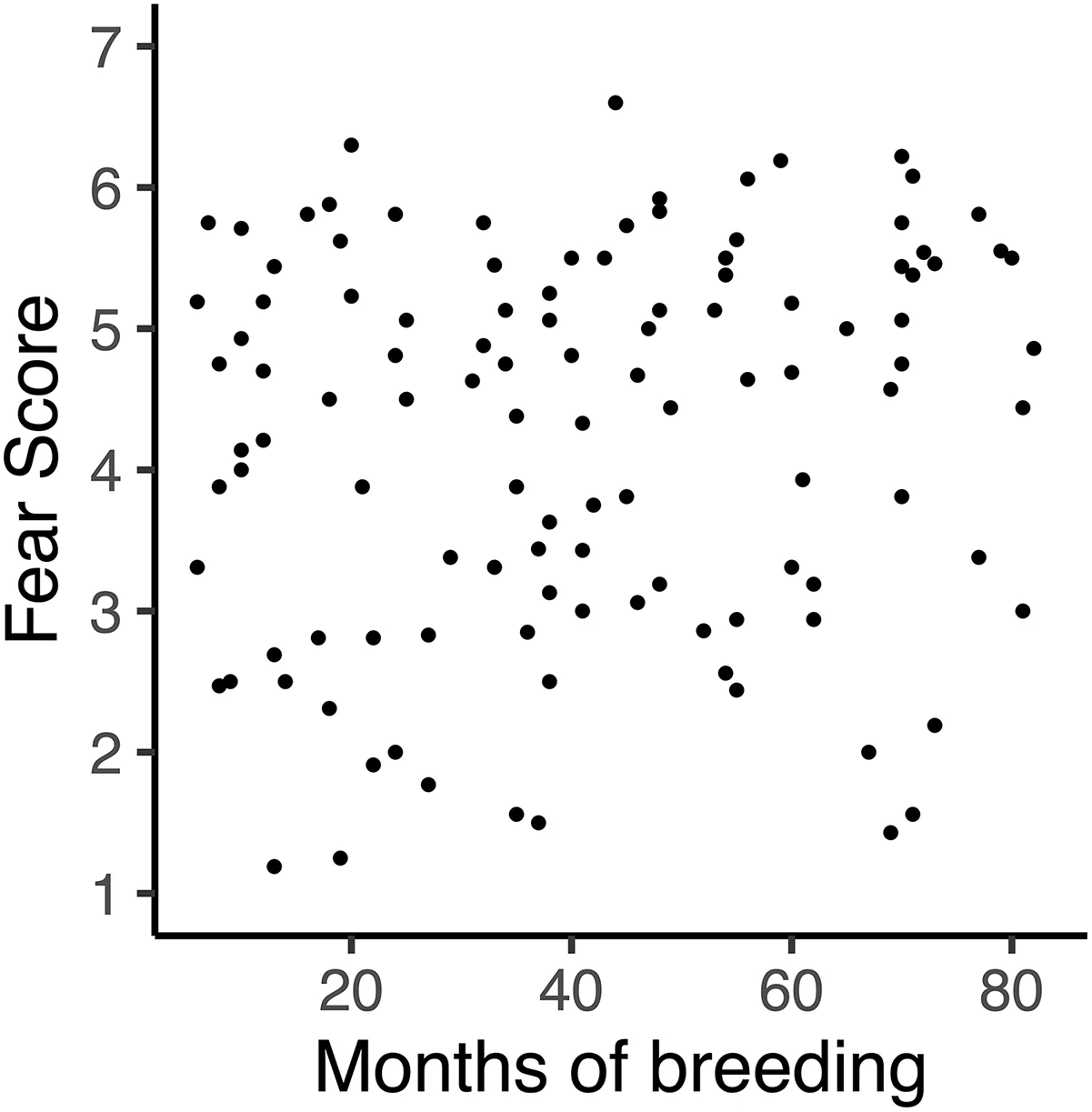
Scatter plot of months of breeding and fear scores (r = 0.134, p = 0.154) in males born as strays (n = 114).

**Fig 9:**
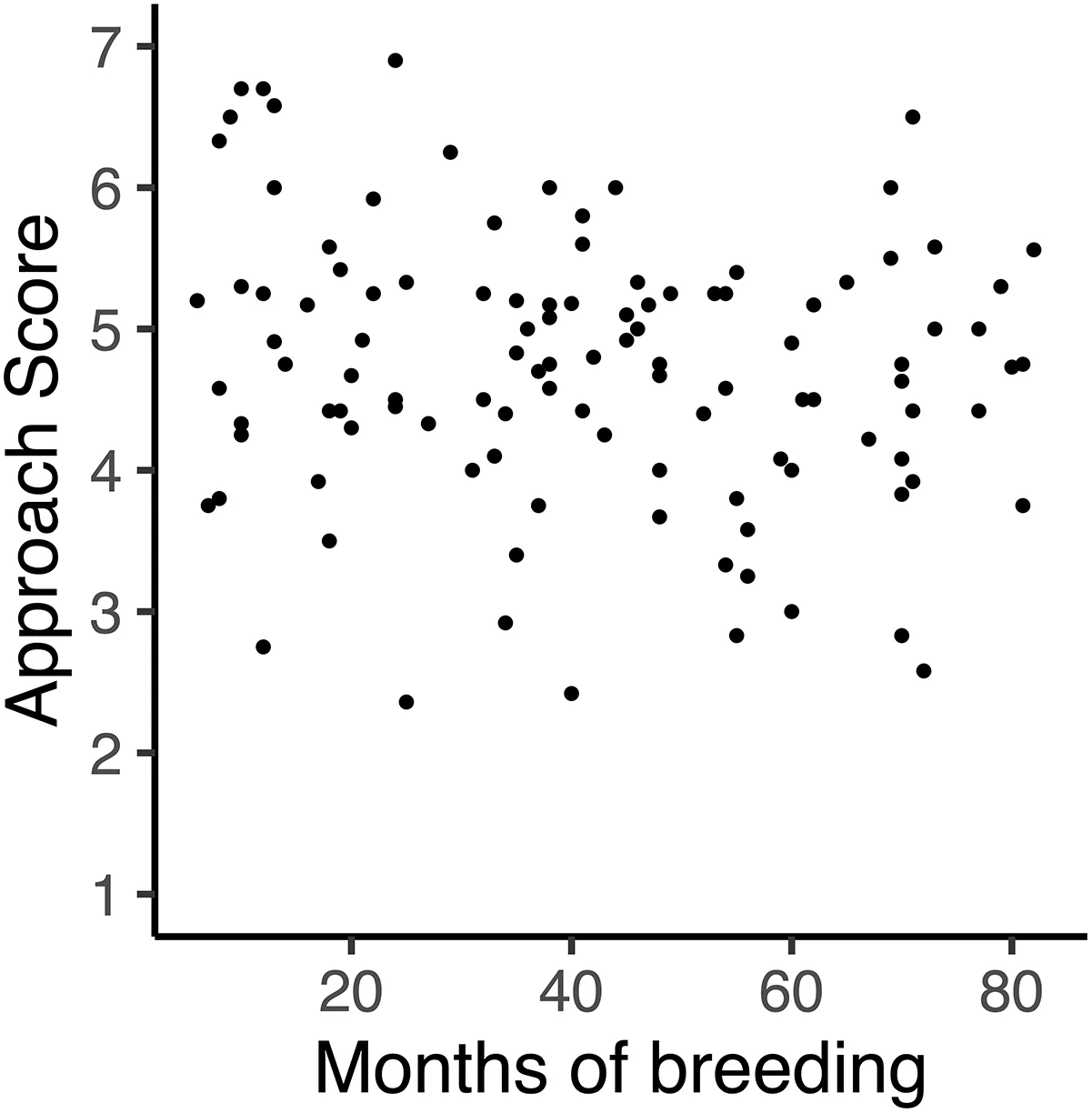
Scatter plot of months of breeding and approach scores (*r* = 0.154, *p* = 0.101).

**Fig 10:**
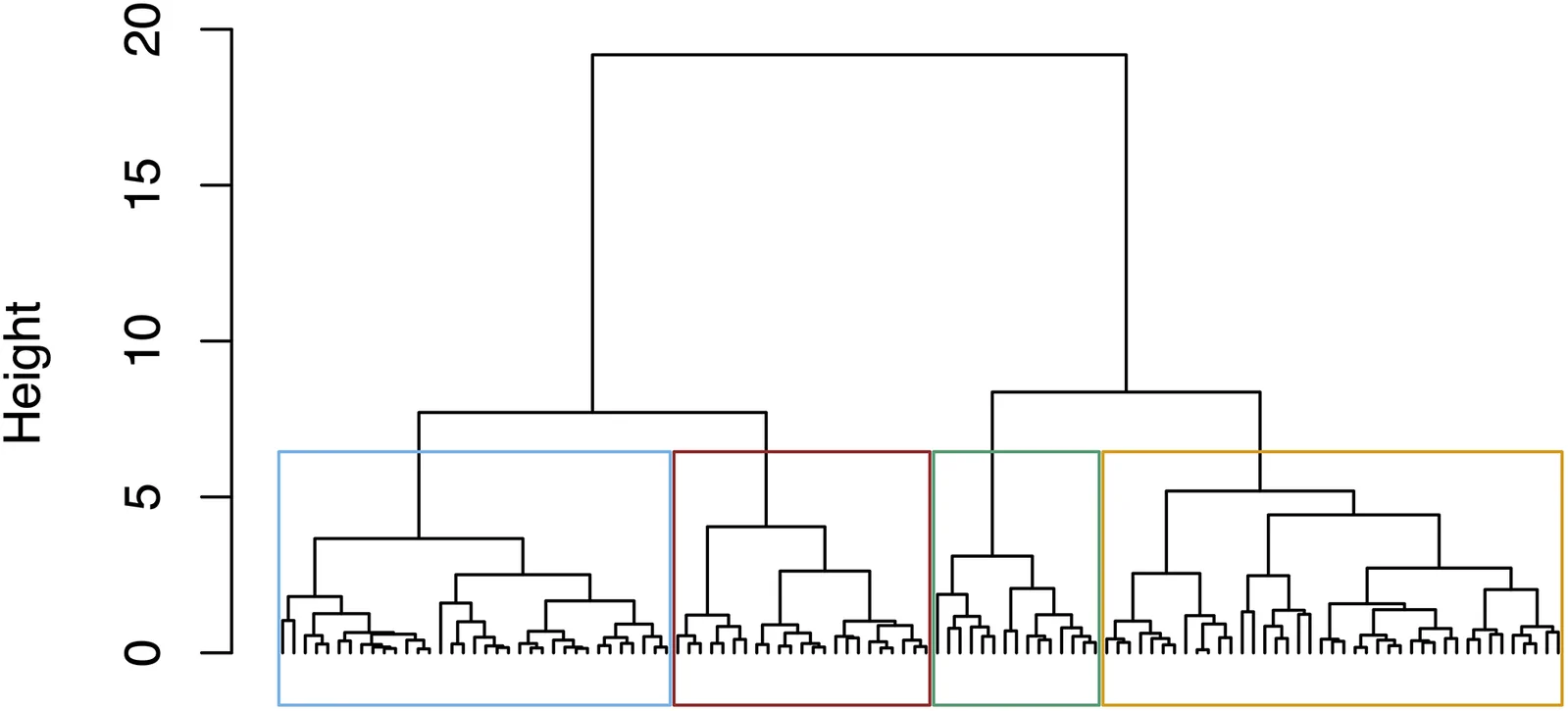
Results of the cluster analysis. The personality traits of male cats who were born stray can be divided into four distinct clusters.

### Discussion

This study aimed to investigate the effect of human interactions on the characteristics of stray male cats. Initially, we postulated that such cats inherently display a human infant-like shyness, prevalent fear behavior, alongside high-approach behavior (Hypothesis 1). Although the overall cluster structure was similar to that observed in Study 2, contrary to our expectations, the number of human infant-like shyness type (Cluster 3, n = 35), characterized by high fear and approach, was less than the other type. Furthermore, we developed two sub-hypotheses regarding the developmental changes in these cats after their adoption by human caregivers (Hypotheses 2–1 and 2–2). Hypothesis 2−1 suggested that after adoption, stray-born male cats would transition to a more sociable type, showing a reduction in fear behaviors while maintaining high approach behaviors. However, our findings revealed no such correlation, suggesting that the impact of human interaction over time on fear and approach behaviors may be less predictable or perhaps influenced by other unmeasured factors. Additionally, Hypothesis 2–2 proposed a bifurcation in post-adoption development, whereby cats would become either more sociable or more fearful. However, these changes were not confirmed by the data. Finally, further analysis confirmed that the data can be divided into four clusters, one of which exhibits the human infant-like shyness type. Cat age differed significantly among the clusters overall, although no pairwise differences remained significant after correction, while months of breeding did not significantly differ among the clusters. These findings should therefore be interpreted cautiously. Similar results were observed in Study 2, suggesting that some cats may have human infant-like shyness, which shows mixed fear and approach behaviors.

## General discussion

We observed that certain cats displayed “human infant-like shyness,” a trait defined by high fear and approach scores, similar to the shyness observed in human infants. In our first study, we identified a correlation between shyness and fear scores, which mirrors findings in human infants. However, in contrast to human infants, no quadratic relationship was evident between shyness and approach scores in cats. Similarly, the quadratic term was not statistically significant in male cats. Subsequent investigations focusing on male cats with increased sample sizes revealed consistent correlations between shyness and fear scores; however, no quadratic relationship with approach scores was observed. Our exploratory analysis, employing cluster analysis, identified a subset of cats exhibiting the human infant-like shyness type, characterized by simultaneous high fear and approach scores. This personality type was more frequently observed in males descended from stray cats. In our third study, we found no evidence suggesting that stray cats transitioned from human infant-like shyness to more sociable or fearful types through human interactions.

These findings do not support the hypothesis that cat shyness comprises a mixture of fear and approach tendencies as observed in human infants [4]. However, the exploratory cluster analyses identified a subset of male cats showing a high-fear/high-approach pattern that may be consistent with human infant-like shyness. This implies that personality traits such as shyness may also exist in other animals, as highlighted by Darwin [8]. Our study demonstrates the diversity of personality traits in cats and suggests that more detailed characterization is needed to understand individual differences in cat personality. The observed variability, similar to that observed in humans, represents a significant insight for understanding animal behavior.

Furthermore, the distinctiveness of human infant-like shyness in certain cats supports our hypothesis that personality traits in animals are as multifaceted as those in humans. The fact that we could measure traits such as shyness in cats using an adaptation of a human questionnaire demonstrates that tools from human personality studies may offer insights into animal personalities. Although cat personality assessments lack standardization [19], our method provides a replicable approach for investigating human infant-like behaviors in cats.

In contrast to our hypothesis, no evidence exists that stray cats transition from complex shyness to more sociable or fearful behaviors through human interaction. Although genetic factors and early life interactions influence sociability [28], it remains uncertain whether these traits are modifiable through subsequent human interactions. The observation of younger human infant-like personality types in male cats in Study 2 suggests the potential for change, yet no such alterations were noted in male cats born as strays in Study 3. Given that human behavior can significantly influence cat behavior [29], future longitudinal studies should elucidate the interactions between human contact and personality changes, extending beyond genetic and early environmental influences.

The results of our questionnaire survey, completed by cat owners, indicate that our method has certain limitations. Our questionnaire survey of cat owners, rather than direct behavioral experiments or genetic analyses, may not fully capture the nuances of cat personalities. Owner-reported questionnaires can capture behavioral tendencies across everyday situations. However, assessments of fear and approach reflect owners’ interpretations of behavior rather than direct measures of cats’ internal motivational states. A more comprehensive understanding can be achieved by observing the behavior of cats, both owners and strangers. In addition, although the CCTI and IBQ-R were originally developed to assess temperament in human infants and young children, the present study adapted specific items concerning shyness, fear and approach to assess corresponding behavioral tendencies in adult cats. Thus, our use of the CCTI and IBQ-R was intended to provide a framework for examining these specific behavioral dimensions across species, rather than to equate infant temperament with adult cat personality. However, this methodological parallel does not establish that the adapted questionnaires measure psychologically equivalent constructs in human infants and adult cats, and their construct validity in cats requires further evaluation. Future studies incorporating cat-specific personality instruments alongside the adapted CCTI and IBQ-R, as well as behavioral observations, would be important for establishing the convergent validity of these scales in cats. Because shyness and fear scores were strongly correlated, their simultaneous inclusion in the cluster analysis may have influenced the resulting cluster structure, which should be considered when interpreting these exploratory findings. Furthermore, data on the birth environment of stray cats, based on owner reports alone, may not be precise. In future research, it will be necessary to clarify the details of birth circumstances or conduct experiments on individuals whose births are known.

The results of our study indicate that despite exhibiting fear, certain cats engage in approach behaviors. This finding suggests that progressively increasing human interactions may facilitate the swift adjustment of cats to the presence of humans. Furthermore, a deeper understanding of individual fear levels and other personality traits in cats could enable shelters and owners to tailor their environments to better accommodate these specific traits. Consequently, this would enhance our overall understanding of cat personalities and lead to improved practices among cat owners and shelter workers, optimizing welfare and interaction strategies.

## Supporting information

S1 File Profiles and behavioral scores of cats included in Studies 1–3. The Excel file contains three worksheets: S1, profiles of cats in Study 1 (n = 82); S2, profiles of male cats in Study 2 (n = 114); and S3, profiles of male cats born as strays in Study 3 (n = 114).
(XLSX)
