# Supplementary material for: Exploration of complex shyness and the relationship between fear and approach behaviors in domestic cats (Felis catus)
Source: PLoS One. 2026 Nov 4;21(11):e0360035. doi: 10.1371/journal.pone.0360035 (PMC13734479; doi:10.1371/journal.pone.0360035)
Supplement: Supplementary file 1 [file pone.0360035.s003.docx]

9th September, 2026

Dr. Brittany N. Florkiewicz,

Academic Editor

PLOS ONE

**Dear Editor:**

On behalf of my co-authors, I wish to re-submit the manuscript titled “Exploration of complex shyness and the relationship between fear and approach behaviors in domestic cats (Felis catus)” The manuscript ID is PONE-D-26-29153.

The manuscript has been rechecked and appropriate changes have been made in accordance with the reviewers’ suggestions. The revised text in our manuscript is visible as tracked changes. The responses to the comments have been prepared and attached herewith. Our responses to comments are in red.

We thank you and the reviewers for your thoughtful suggestions and insights, which have enriched the manuscript and produced a better and more balanced account of the research. We hope that the revised manuscript is now suitable for publication in your journal.

Thank you for your consideration. I look forward to hearing from you.

Sincerely,

Dr. Madoka Hattori

Kyoto University, Kyoto, Japan

E-mail address: hattorimadoka@gmail.com

**Additional Editor Comments:**

In response to your request regarding the funding disclosure and the role of the funders, we would like to provide the following amended statement:

This study was financially supported by JST SPRING (JPMJSP2110 to M.H.), the Japanese Society of Feline Medicine (JSFM to M.H.), JSPS KAKENHI (24H02200 to S.Y.), and JST FOREST (JPMJFR221I to S.Y.). The funders had no role in study design, data collection and analysis, decision to publish, or preparation of the manuscript.

In accordance with the journal’s instructions, we have also removed the funding-related text from the Acknowledgments section of the revised manuscript. Thank you for your consideration.

**Reviewers’ comments:**

**Response to Reviewers:** We sincerely thank the reviewers for their careful evaluation of our manuscript and for their constructive and helpful comments. We have revised the manuscript accordingly and addressed each comment point by point below. All changes made in response to the reviewers’ comments are indicated in the revised manuscript. Line numbers cited in our responses refer to the clean revised manuscript.

**Reviewer #1’ comments:**

**Please consider the following points:-**

**Response:** We thank the reviewer for the careful evaluation of our manuscript and for the constructive comments. We have revised the Introduction to provide a clearer behavioral definition of complex shyness and to explain the theoretical rationale underlying our original hypothesis, as detailed below.

1. **Behavioral Definition:** What specific, observable feline behaviors would represent this "complex shyness" in a daily setting? For instance, how does a cat with "high fear and high approach" behave differently from a purely "sociable" or purely "fearful" cat?

**Response:** Thank you for this comment. We have expanded the Introduction to define complex shyness in behavioral terms. For example, when an unfamiliar person enters the cat’s home, a cat with high fear and high approach tendencies may initially retreat but continue to monitor the person from a distance and gradually emerge or move closer. This hypothesized pattern differs from that of a sociable cat, which may approach readily with little initial avoidance, and from that of a fearful cat, which may remain hidden and show little tendency to approach. We have added this behavioral example to the Introduction (Lines 103–108).

2. **Theoretical Mechanism:** Please elaborate on the psychological or evolutionary mechanism behind the U-shaped quadratic curve. Why would cats with moderate shyness scores decrease their approach behaviors, while cats with extreme shyness scores (very high) still display approach? Providing a clearer theoretical bridging from human infant literature [4] to feline behavioral ecology would significantly strengthen the rationale of this study.

**Response:** Thank you for this important comment. We have clarified the theoretical basis of the predicted relationship. Based on the human infant literature [4], we hypothesized that fear and approach tendencies may coexist in highly shy individuals. Because neither preverbal infants nor cats can verbally describe this conflict, it must be inferred from observable behavior or caregiver reports. We applied this framework to cats as a working hypothesis. We have revised the Introduction to clarify this theoretical rationale (Lines 101–112).

**Reviewer #2’ comments:**

**Response:** We thank the reviewer for the careful and constructive comments. We agree that the manuscript required clearer justification for the adapted questionnaires and a stricter distinction between confirmatory and exploratory analyses. We have revised the manuscript accordingly and moderated the interpretation of the cluster analyses.

**Major comments**

1. **Justification for adapting human infant questionnaires to adult cats**

The most important concern is the use of the Colorado Childhood Temperament Inventory (CCTI) and the Infant Behavior Questionnaire-Revised (IBQ-R) for adult domestic cats. The Introduction clearly explains why the authors wished to test the same theoretical framework that has been applied to human infants. However, using the same theoretical framework does not automatically justify using the same measurement instruments. The manuscript states that the questionnaires were "adapted" for cats by modifying several items. However, no theoretical or psychometric justification is provided for assuming that the underlying constructs remain equivalent after such modifications. Internal consistency (Cronbach's alpha) demonstrates reliability but does not establish construct validity. I therefore recommend that the authors discuss more explicitly:

* why human infant temperament questionnaires are theoretically appropriate for adult cats,

* how the construct validity of the adapted questionnaires can be justified,

* and what limitations should be acknowledged regarding this adaptation.

**Response:** We thank the reviewer for this important comment. We selected the CCTI and IBQ-R because they separately assess shyness, fear, and approach, which were required to test the approach–avoidance hypothesis derived from the human infant literature. Our adaptation was also based on a methodological parallel between the two research settings. In human infant research, behavioral tendencies expressed in relation to familiar caregivers and unfamiliar individuals are assessed by asking an adult caregiver to report the infant’s behavior. In the present study, we applied the same proxy-report structure to the cat–human relationship: an adult owner reported the cat’s observable behavior in relation to its owner, unfamiliar people, and novel situations. Thus, in both settings, an adult who knows the nonverbal subject well evaluates its behavior across everyday social contexts. This parallel provides a rationale for adapting the questionnaires to cats, although it does not imply that the underlying psychological constructs or attachment processes are identical in human infants and adult cats. We have added this methodological rationale to the revised manuscript and have also addressed the remaining issue of construct validity for the adapted CCTI and IBQ-R in the Limitations section (Lines 114-125 and 590-603).

2. **Why were existing feline personality instruments not used?**

The manuscript itself introduces several feline-specific personality assessments, including the Domestic Cat Personality Inventory, Fe-BARQ, and other established instruments. However, the manuscript does not adequately explain why these existing feline personality questionnaires were not used, either as primary measures or for convergent validation of the adapted CCTI and IBQ-R. This omission weakens the methodological justification of the study. At minimum, the authors should explain why these existing instruments were considered unsuitable for testing their hypothesis, or discuss the limitations of relying exclusively on adapted human questionnaires.

**Response:** We appreciate the reviewer’s suggestion. Existing feline-specific instruments assess broad personality and behavioral traits but do not separately measure shyness, fear, and approach in the form required for our hypothesis. We therefore used the CCTI and IBQ-R to maintain correspondence with the human infant framework. We have clarified this rationale and acknowledged the lack of convergent validation as a limitation. (Lines 85-98 and 598-600).

3. **Temperament versus personality trait**

Another conceptual issue concerns the developmental stage of the subjects. IBQ-R was originally developed to assess temperament during infancy, whereas the present study investigates adult cats (1–7 years old). In developmental psychology, temperament and mature personality traits are conceptually distinct constructs. The manuscript would benefit from a discussion explaining why an infant temperament framework is appropriate for assessing stable behavioral tendencies in adult cats. Without such discussion, readers may question whether the constructs being measured correspond to those originally intended by the questionnaire.

**Response:** Thank you for this important comment. We agree that temperament in human infants and mature personality traits in adult cats should not be regarded as conceptually equivalent. Our intention was not to use the IBQ-R as a general personality measure for adult cats, but to adapt specific IBQ-R items assessing fear and approach tendencies, which were central to our hypothesis concerning complex shyness. We have clarified this rationale in the manuscript and added this issue to the Discussion as a limitation of the cross-species adaptation (Lines 99-125 and 590-614).

4. **Logical progression across Studies 1–3**

The overall structure of the manuscript could be clarified.

Study 1 proposes a clear confirmatory hypothesis based on previous findings in human infants.

Study 2 appropriately attempts to replicate the findings with a larger sample of male cats.

However, the primary confirmatory hypothesis—the predicted quadratic relationship between shyness and approach—was not supported in either Study 1 or Study 2. After these negative findings, the manuscript shifts its emphasis to hierarchical cluster analysis, an exploratory analysis, and subsequently interprets one cluster as representing "human infant-like shyness." Although exploratory analyses are valuable for generating new hypotheses, they should be clearly distinguished from confirmatory analyses. I therefore recommend that the authors explicitly distinguish between hypothesis-testing analyses and hypothesis-generating analyses throughout the manuscript and moderate conclusions derived from exploratory findings.

**Response:** We agree that the original manuscript did not sufficiently distinguish confirmatory and exploratory analyses. We have revised the Abstract and General Discussion to clarify that the predicted quadratic relationship was not supported, whereas the subsequent cluster analyses were exploratory and hypothesis-generating. We have also moderated the interpretation of these exploratory findings(Lines 23-38 and 558–566).

5. **Interpretation of the cluster analysis**

The hierarchical cluster analysis itself is acceptable as an exploratory method. However, I am concerned that the conclusions may overstate what this analysis demonstrates. Cluster analysis is an unsupervised exploratory technique. It suggests patterns within the observed data but does not establish the existence of objectively distinct personality phenotypes. Expressions such as "identified four personality phenotypes" appear stronger than warranted by the evidence. More cautious wording (e.g., "suggested four clusters" or "identified four candidate personality profiles") would better reflect the exploratory nature of the analysis. Furthermore, the clustering variables include shyness and fear scores that are themselves highly correlated. The authors should discuss the possible influence of correlated variables on the clustering solution.

**Response:** Thank you for this important comment. We agree that hierarchical cluster analysis is exploratory and does not establish objectively distinct personality phenotypes. We have therefore revised the wording to “candidate personality phenotypes” and moderated the interpretation of the clustering results. We have also added a statement to the General Discussion acknowledging that the strong correlation between shyness and fear scores may have influenced the clustering solution (Lines 31–34 and 601–603).

6. **Statistical interpretation**

Overall, the statistical methods selected for the confirmatory hypotheses (Spearman correlations and quadratic regression) are appropriate. However, the interpretation of the statistical results deserves more careful consideration. In both Study 1 and Study 2, the quadratic relationship between shyness and approach was not statistically significant. Therefore, the primary hypothesis was not supported. Subsequent exploratory analyses should not be interpreted as providing equivalent evidence for the original hypothesis. I recommend that the authors more clearly separate the evidence supporting the original hypothesis from exploratory observations that require future independent replication.

**Response:** Thank you for this comment. We agree that the exploratory findings should not be interpreted as equivalent evidence for the original hypothesis. As also addressed in response to Comment 4, we have revised the Abstract and General Discussion to explicitly state that the primary confirmatory hypothesis was not supported and to distinguish the subsequent exploratory findings (Lines 29–38 and 549-552).

**Minor comments**

1. In the Methods section (Study 1), the manuscript uses the first-person singular expression "I changed..." when describing questionnaire modification. This appears inconsistent with the rest of the manuscript and is likely an editorial oversight.

**Response:** Thank you for pointing this out. We have corrected the first-person singular expression to ensure consistency with the rest of the manuscript (Line 169-171).

2. The manuscript would benefit from a more explicit discussion of the limitations of owner-reported questionnaires, particularly when assessing internal motivational states such as approach and fear.

**Response:** We agree and have expanded the limitations section to clarify that owner-reported questionnaires reflect interpretations of observable behavior rather than direct measurements of internal motivational states (Lines 586–588).

3. If feasible, reporting additional effect size measures and discussing the robustness of the cluster solution (e.g., through alternative clustering validation methods) would further strengthen the manuscript.

Response: Thank you for this suggestion. In response to this and another reviewer’s comments, we conducted additional comparisons among the clusters (Lines 349–355).

**Reviewer #3’ comments:**

**Response:** We thank the reviewer for the careful and constructive assessment. We agree that the rationale connecting Studies 1–3 was not sufficiently explained and that some descriptive observations were interpreted too strongly. We have revised the manuscript accordingly and moderated the interpretation of the cluster analyses.

**Line 24:** Please clarify that the numbers reported refer to the number of cats included in Studies 1, 2, and 3, respectively. At this point in the manuscript, it is also difficult for readers to understand why Study 2 focused exclusively on male cats and why Study 3 was further restricted to stray-born male cats. A brief explanation or cross-reference would improve the readability of the manuscript.

**Response:** Thank you for this helpful comment. We have revised Line 24 to explicitly clarify that the three sample sizes correspond to Studies 1, 2, and 3, respectively. We have also briefly explained that Study 2 focused on male cats to follow up on the sex-specific pattern observed in Study 1, whereas Study 3 focused on stray-born male cats to further examine the pattern identified among stray-born cats in Study 2 (Lines 23–29).

**Line 32, 35:** Please replace "feline" with "cat" throughout the manuscript for consistency.

**Response:** Thank you for this comment. We have replaced “feline” with “cat” throughout the manuscript for consistency (Lines 39, 612).

**Line 33:** Could you clarify why the results of the cluster analysis support the conclusion that shyness is predominantly associated with fear?

**Response:** Thank you for this important comment. We agree that the cluster analysis itself does not demonstrate that shyness is predominantly associated with fear. Rather, the close association between shyness and fear is supported by the strong positive correlations observed between these scores, whereas the exploratory cluster analyses identified a subset of cats with both high fear and high approach scores. We have revised the Abstract to distinguish these two findings more clearly by specifying the analysis supporting each conclusion (Lines 34–38).

**Line 71:** Please italicize “*Felis catus”* throughout the manuscript.

**Response:** Thank you for this comment. We have italicized the scientific name “*Felis catus*” throughout the manuscript for consistency (Lines 75).

**Line 73:** Is the term "semi-domestication" commonly used in literature?

**Response:** Thank you for this comment. The term “semi-domesticated” has been used in the literature to describe domestic cats, reflecting the relatively limited human control over their breeding compared with that of many other domesticated species. However, we agree that the original wording could imply that “semi-domestication” represents a universally accepted classification. We have therefore revised the sentence to clarify that cats have been described as “semi-domesticated” in the literature (Lines 76–82).

**Line 75:** Please provide the references supporting the statement "earlier research."

**Response:** Thank you for this comment. We have added references (Bradshaw, 2016) supporting the statement regarding differences in the domestication histories and social behavior of cats and dogs. We have also revised the sentence to remove the statement regarding wider variation in cat personality traits, for which direct comparative evidence is limited, and to more accurately reflect the findings of the cited literature (Lines 79–82).

**Line 80:** Please provide the references supporting the statement "earlier studies."

**Response:** Thank you for this comment. We have revised this section to clarify that previous studies of cat personality have examined a variety of personality traits other than shyness, and have explicitly cited examples of these previous studies [18,19] (Lines 85–98).

**Line 145-146:** Can "going shopping" for human infants be considered equivalent to "going to the hospital" for cats?

**Response:** Thank you for pointing this out. The statement that “going shopping” was changed to “going to the hospital” did not accurately describe the item used in the actual survey. During manuscript preparation, provisional wording was used because the complete questionnaire items could not be reproduced owing to copyright restrictions, and this wording was inadvertently retained in the submitted manuscript. We have therefore corrected the text to describe an item adaptation that was actually used in the survey. Specifically, “Quickly becomes friendly with other children” was modified to “Quickly becomes friendly with familiar people (non-family members with whom the cat has interacted several times).” This adaptation retained the original item’s focus on becoming friendly with familiar social partners. The manuscript has been revised accordingly (Lines 169–171).

**Line 150, 151:** Could you clarify whether the items "My cat makes friends easily" and "My cat is very sociable" refer to interactions with humans or with other cats? The intended target of these questions is unclear, and different respondents may have interpreted them differently. Please clarify the intended context and how respondents were instructed to answer these items.

**Response:** Thank you for this comment. All items in this questionnaire referred to the cats’ behavior toward humans. At the beginning of the questionnaire, respondents were explicitly instructed to answer the questions based on how their cats behaved toward humans. Therefore, “My cat makes friends easily” and “My cat is very sociable” were intended to refer to interactions with humans rather than with other cats. To preserve the wording of the items actually administered to respondents, we did not modify the individual items. Instead, we added the introductory instruction to the Methods to clarify the context in which respondents answered these items(Lines 172–178).

**Line 181-183:** Please clarify the rationale for using a regression model with a quadratic term. It would be helpful to explain why a quadratic relationship was hypothesized.

**Response:** Thank you for this comment. The quadratic term was included to test our hypothesis that the relationship between shyness and approach scores would be nonlinear, with the association differing across low, intermediate, and high levels of shyness rather than following a simple linear pattern. This hypothesis was based on the proposed coexistence of fear and approach tendencies at high levels of shyness. We have clarified this hypothesis and its theoretical rationale in the Introduction, as well as the rationale for including the quadratic term in the Statistical Analysis section(Lines 101-112 and 208–211).

**Line 199-207:** The authors should be clarifying the rationale for conducting separate analyses for male and female cats. Do the authors hypothesize that different mechanisms underlie shyness in male and female cats? Even if these analyses were exploratory, some theoretical or empirical background should be provided to explain why sex differences were considered relevant and why sex-specific analyses were performed.

**Response:** Thank you for this helpful comment. We did not have an a priori hypothesis that different mechanisms underlie shyness in male and female cats. However, previous research has reported sex differences in several behavioral traits in cats, including nervousness (Takeuchi & Mori, 2009). We have added this background and clarified that the sex-specific analyses were exploratory(Lines 216–219).

**Line 208-215:** The low statistical power should not be attributed solely to the limited sample size. Given that the observed effect size was also relatively small, an alternative explanation is that the quadratic relationship is genuinely weak or absent in male cats. This possibility should be considered and discussed alongside the explanation of insufficient statistical power.

**Response:** Thank you for this important comment. We agree that the nonsignificant result in male cats should not be attributed solely to the limited sample size, because the quadratic relationship may in fact be weak or absent. However, the possibility that the relationship was not detected because of the small number of male cats in Study 1 could not be excluded. We have revised the Discussion to clarify that Study 2 was conducted with an expanded sample of male cats to examine these alternative explanations (Lines 250–254).

**Line 282:** Please clarify the rationale for conducting the cluster analysis. It is currently unclear what specific research question this analysis was intended to address and how it relates to the overall hypothesis of the study.

Response: Thank you for this comment. We have clarified the rationale for the exploratory cluster analysis. Specifically, the analysis was conducted to examine whether distinct groups characterized by different combinations of shyness, fear, and approach could be identified, particularly a subgroup showing both high fear and high approach tendencies as predicted by the complex shyness hypothesis. We have added this rationale to the Statistical analysis section (Lines 313–320).

**Line 283:** Please clarify how the comparisons among the clusters were conducted. Were the conclusions based solely on descriptive statistics? Given the sample size, it would be more appropriate to statistically compare the clusters (e.g., for age, birth background, or other relevant variables) to support the interpretations presented in the Results and Discussion.

Response: We thank the reviewer for this helpful suggestion. In the original manuscript, the comparisons of age and childhood background among clusters were based on descriptive statistics. Following the reviewer’s suggestion, we conducted additional post hoc comparisons among the four clusters. Cat age did not significantly differ among clusters (Kruskal–Wallis test, χ²(3) = 1.54, p = .673), and childhood background was not significantly associated with cluster membership (Fisher’s exact test, p = .201). As these analyses were not included in our preregistration, we have explicitly identified them as post hoc analyses in the revised manuscript(Lines 349-350, 354–355 and 497-502).

**Line 318-319:** The statement that the higher proportion of stray-born cats in Cluster 3 "was closer to the hypothesis" is difficult to understand. The manuscript does not appear to present an a priori hypothesis regarding the distribution of stray-born cats across clusters. Please clarify which hypothesis this result supports

**Response:** Thank you for this comment. We agree that there was no a priori hypothesis regarding the distribution of stray-born cats across clusters. We have therefore removed the statement that this result “was closer to the hypothesis” and revised the text accordingly (Line 356).

**Line 386-388:** Could you clarify why they hypothesized that this pattern would be observed specifically in male cats? The biological or behavioral rationale for focusing on males is currently unclear. If there is evidence suggesting that the mechanisms underlying shyness differ between male and female cats, this should be described and appropriately referenced.

**Response:** Thank you for this comment. We did not have an a priori hypothesis that the mechanisms underlying shyness differ between male and female cats. Study 2 focused on male cats because the exploratory sex-specific analysis in Study 1 showed a positive association between shyness and approach in male cats, but not in female cats. We have revised the text to clarify that the focus on male cats in Study 2 was based on this finding from Study 1 rather than on a prior hypothesis of sex-specific mechanisms. We have also clarified that Study 3 focused on stray-born male cats because Study 2 identified the Human infant-like shyness type among cats with this natal background (Lines 425–432).

**Line409:** Study 2 and Study 3 investigate different hypotheses using different statistical analyses. Therefore, it is unclear why the sample size calculated for Study 2 was directly applied to Study 3. If the primary outcome and statistical approach differed, a study-specific sample size justification would seem more appropriate.

**Response:** Thank you for this comment. As preregistered, the target sample size for Study 3 was set at 114, following the sample size used in Study 2. We agree that the sample size calculation for Study 2 does not directly justify the sample size for the different analyses conducted in Study 3. We have therefore clarified the preregistered basis for the sample size and additionally conducted a sensitivity power analysis for the correlation analysis in Study 3. We have therefore clarified that the target sample size followed the preregistered design and was not based on a study-specific power analysis (Lines 455-457).

**Line 443-446:** Statistical comparisons among the clusters should be conducted. Given that the manuscript discusses differences in variables such as age and birth background across clusters, these differences should be formally tested using appropriate statistical methods rather than relying solely on descriptive statistics.

**Response:** We thank the reviewer for this helpful suggestion. Following the reviewer’s suggestion, we conducted additional statistical comparisons among the four clusters. Cat age differed significantly among the clusters (Kruskal–Wallis test, χ²(3) = 8.25, p = .041); however, post hoc pairwise comparisons with Holm correction showed no significant differences between individual clusters (all adjusted *p* > .05). The number of months of breeding did not significantly differ among the clusters (Kruskal–Wallis test, χ²(3) = 7.35, *p* = .062). These analyses were not specified in the preregistration and are therefore reported as additional exploratory analyses. We have added these analyses to the Methods and Results sections and revised the Discussion accordingly (Lines 475-478, 497–502 and 521-524).

**Line 491-492:** Could you clarify the basis for the statement that a "potential quadratic relationship" was observed in male cats? As the quadratic term was not statistically significant, it is unclear which results support this interpretation.

**Response:** Thank you for this comment. We agree that the term “potential quadratic relationship” was not sufficiently supported because the quadratic term was not statistically significant in male cats. We have therefore removed this interpretation and revised the text accordingly (Lines 549–552).

**Line 505:** Despite common perception of uniform behavior in domestic animals; Please provide a reference to support the statement.

**Response:** Thank you for this comment. We agree that this statement was not sufficiently supported. We have therefore removed the statement regarding common perceptions of uniform behavior in domestic animals and revised the text accordingly (Lines 563–566).

We thank the reviewers again for their constructive comments and hope that the revised manuscript satisfactorily addresses all concerns raised.
