# Supplementary material for: Exploration of complex shyness and the relationship between fear and approach behaviors in domestic cats (Felis catus)
Source: PLoS One. 2026 Nov 4;21(11):e0360035. doi: 10.1371/journal.pone.0360035 (PMC13734479; doi:10.1371/journal.pone.0360035)
Supplement: Peer review history

# Peer review: Decision Letter 0

**DOI:** 10.1371/journal.pone.0360035.r001

**Authors:** Brittany Florkiewicz

15 Jul 2026

Dear Dr. Hattori,

Thank you for submitting your manuscript for consideration in PLOS One. I agree with all three reviewers: this study addresses an original question and provides a unique perspective on cat behavior. However, all three reviewers have recommended revisions to the theoretical and methodological framework of your study. I agree with their suggestions, and believe implementing these changes will enhance the strength and transparency of your research, ultimately making it more suitable for publication. I look forward to reading your revised manuscript!

Please submit your revised manuscript by Sep 13 2026 11:59PM. If you will need more time than this to complete your revisions, please reply to this message or contact the journal office at plosone@plos.org. When you're ready to submit your revision, log on to https://www.editorialmanager.com/pone/ and select the 'Submissions Needing Revision' folder to locate your manuscript file.

- A letter that responds to each point raised by the academic editor and reviewer(s). You should upload this letter as a separate file labeled 'Response to Reviewers'.
- A marked-up copy of your manuscript that highlights changes made to the original version. You should upload this as a separate file labeled 'Revised Manuscript with Track Changes'.
- An unmarked version of your revised paper without tracked changes. You should upload this as a separate file labeled 'Manuscript'.

If you would like to make changes to your financial disclosure, please include your updated statement in your cover letter. Guidelines for resubmitting your figure files are available below the reviewer comments at the end of this letter.

If applicable, we recommend that you deposit your laboratory protocols in protocols.io to enhance the reproducibility of your results. Protocols.io assigns your protocol its own identifier (DOI) so that it can be cited independently in the future. For instructions see: https://journals.plos.org/plosone/s/submission-guidelines#loc-laboratory-protocols. Additionally, PLOS ONE offers an option for publishing peer-reviewed Lab Protocol articles, which describe protocols hosted on protocols.io. Read more information on sharing protocols at https://plos.org/protocols?utm_medium=editorial-email&utm_source=authorletters&utm_campaign=protocols.

As the corresponding author, your ORCID iD is verified in the submission system and will appear in the published article. PLOS supports the use of ORCID, and we encourage all coauthors to register for an ORCID iD and use it as well. Please encourage your coauthors to verify their ORCID iD within the submission system before final acceptance, as unverified ORCID iDs will not appear in the published article. *Only*  the individual author can complete the verification step; PLOS staff *cannot*  verify ORCID iDs on behalf of authors.

We look forward to receiving your revised manuscript.

Kind regards,

Brittany N. Florkiewicz, Ph.D.

Academic Editor

PLOS One

Journal Requirements:

When submitting your revision, we need you to address these additional requirements.

1. Please ensure that your manuscript meets PLOS ONE's style requirements, including those for file naming. The PLOS ONE style templates can be found at

https://journals.plos.org/plosone/s/file?id=wjVg/PLOSOne_formatting_sample_main_body.pdf and

https://journals.plos.org/plosone/s/file?id=ba62/PLOSOne_formatting_sample_title_authors_affiliations.pdf

2. Please provide additional details regarding participant consent. In the ethics statement in the Methods and online submission information, please ensure that you have specified (1) whether consent was informed and (2) what type you obtained (for instance, written or verbal, and if verbal, how it was documented and witnessed). If your study included minors, state whether you obtained consent from parents or guardians. If the need for consent was waived by the ethics committee, please include this information.

If you are reporting a retrospective study of medical records or archived samples, please ensure that you have discussed whether all data were fully anonymized before you accessed them and/or whether the IRB or ethics committee waived the requirement for informed consent. If patients provided informed written consent to have data from their medical records used in research, please include this information.

3. We note that the grant information you provided in the ‘Funding Information’ and ‘Financial Disclosure’ sections do not match.

When you resubmit, please ensure that you provide the correct grant numbers for the awards you received for your study in the ‘Funding Information’ section.

4. Thank you for stating the following financial disclosure: [This study was financially supported by JST SPRING (JPMJSP2110 to M.H.), JSFM Japanese Society of Feline Medicine (JSFM to M.H.), JSPS KAKENHI (24h02200 to S.Y.), and JST FOREST (JPMJFR221I to S.Y.)]

Please state what role the funders took in the study.  If the funders had no role, please state: ""The funders had no role in study design, data collection and analysis, decision to publish, or preparation of the manuscript.""

If this statement is not correct you must amend it as needed.

Please include this amended Role of Funder statement in your cover letter; we will change the online submission form on your behalf.

5. Thank you for stating the following in the Acknowledgments Section of your manuscript: [Acknowledgments The authors would like to thank the cat owners and cats for providing the behavioral assessment data and samples. We also thank Ryoji Nishizawa (Association for Promotion of Animal Welfare and Socialization) for helping with data collection. We thank Dr. Hideki Sugiura, Dr. Kodze Kinoshita, and Dr. Miho Murayama (Kyoto University) for reviewing the paper draft and providing helpful comments. We are grateful to Dr. Yoshitaka Matsuda for providing the Japanese version of the CCTI questionnaire. This study was financially supported by JST SPRING (JPMJSP2110 to M.H.), JSFM Japanese Society of Feline Medicine (JSFM to M.H.), JSPS KAKENHI (24h02200 to S.Y.) and JST FOREST (JPMJFR221I to S.Y.).

We note that you have provided funding information that is not currently declared in your Funding Statement. However, funding information should not appear in the Acknowledgments section or other areas of your manuscript. We will only publish funding information present in the Funding Statement section of the online submission form.

Please remove any funding-related text from the manuscript and let us know how you would like to update your Funding Statement. Currently, your Funding Statement reads as follows: [This study was financially supported by JST SPRING (JPMJSP2110 to M.H.), JSFM Japanese Society of Feline Medicine (JSFM to M.H.), JSPS KAKENHI (24h02200 to S.Y.), and JST FOREST (JPMJFR221I to S.Y.)]

Please include your amended statements within your cover letter; we will change the online submission form on your behalf.

6. When completing the data availability statement of the submission form, you indicated that you will make your data available on acceptance. We strongly recommend all authors decide on a data sharing plan before acceptance, as the process can be lengthy and hold up publication timelines. Please note that, though access restrictions are acceptable now, your entire data will need to be made freely accessible if your manuscript is accepted for publication. This policy applies to all data except where public deposition would breach compliance with the protocol approved by your research ethics board. If you are unable to adhere to our open data policy, please kindly revise your statement to explain your reasoning and we will seek the editor's input on an exemption. Please be assured that, once you have provided your new statement, the assessment of your exemption will not hold up the peer review process.

7. If the reviewer comments include a recommendation to cite specific previously published works, please review and evaluate these publications to determine whether they are relevant and should be cited. There is no requirement to cite these works unless the editor has indicated otherwise.

[Note: HTML markup is below. Please do not edit.]

Reviewers' comments:

Reviewer's Responses to Questions

**Comments to the Author**

1. Is the manuscript technically sound, and do the data support the conclusions?

Reviewer #1: Yes

Reviewer #2: Yes

Reviewer #3: Partly

**********

2. Has the statistical analysis been performed appropriately and rigorously?

Reviewer #1: Yes

Reviewer #2: Yes

Reviewer #3: No

**********

3. Have the authors made all data underlying the findings in their manuscript fully available?

The PLOS Data policy

Reviewer #1: Yes

Reviewer #2: Yes

Reviewer #3: Yes

**********

4. Is the manuscript presented in an intelligible fashion and written in standard English?

Reviewer #1: Yes

Reviewer #2: Yes

Reviewer #3: Yes

**********

Reviewer #1: The manuscript entitled "Exploration of complex shyness and the relationship between fear and approach behaviors in domestic cats (Felis catus)" investigated the conceptual structure of feline shyness by applying the framework of human infant shyness to domestic cats through a series of questionnaire surveys. Although the authors' primary hypothesis (i.e., the quadratic relationship between shyness and approach scores) was statistically rejected, they successfully identified a distinct subset of cats exhibiting a coexistence of fear and approach traits through exploratory cluster analyses.

The scientific transparency and methodological rigor of this study are highly commendable, as evidenced by the consistent use of pre-registration across Studies 1 to 3 and the careful calculation of sample sizes based on power analyses. I believe this manuscript will be well-suited for publication in PLOS ONE once the following crucial points regarding the theoretical rationale and behavioral definitions are addressed. Therefore, I recommend a Minor Revision.

Conceptual Clarification of the "Complex Shyness Hypothesis":

The introduction of the "Complex Shyness Hypothesis" (lines 95–96) and its predicted statistical representation (lines 109–114) lacks sufficient conceptual and behavioral explanation. While the authors state that shyness involves a combination of fear and approach traits, it is difficult for readers to intuitively understand why this combination manifests specifically as a "quadratic relationship" where high approach scores occur at both extremely low and extremely high shyness scores.

To improve the manuscript, please address the following:

Behavioral Definition: What specific, observable feline behaviors would represent this "complex shyness" in a daily setting? For instance, how does a cat with "high fear and high approach" behave differently from a purely "sociable" or purely "fearful" cat?

Theoretical Mechanism: Please elaborate on the psychological or evolutionary mechanism behind the U-shaped quadratic curve. Why would cats with moderate shyness scores decrease their approach behaviors, while cats with extreme shyness scores (very high) still display approach? Providing a clearer theoretical bridging from human infant literature [4] to feline behavioral ecology would significantly strengthen the rationale of this study.

Reviewer #2: I would first like to commend the authors for addressing an original and thought-provoking question. The attempt to investigate whether the concept of "complex shyness," originally proposed in human infancy, can be extended to domestic cats is novel and has the potential to stimulate interdisciplinary discussion between comparative psychology, animal behavior, and developmental psychology. The manuscript is generally well written, and the authors should be congratulated for conducting three sequential studies to examine this challenging hypothesis.

However, I have several concerns regarding the theoretical rationale, the psychometric validity of the measurements, and the interpretation of the statistical analyses. I believe these issues should be addressed before the manuscript can be considered for publication.

### Major comments

**1. Justification for adapting human infant questionnaires to adult cats**

The most important concern is the use of the Colorado Childhood Temperament Inventory (CCTI) and the Infant Behavior Questionnaire-Revised (IBQ-R) for adult domestic cats.

The Introduction clearly explains why the authors wished to test the same theoretical framework that has been applied to human infants. However, using the same theoretical framework does not automatically justify using the same measurement instruments.

The manuscript states that the questionnaires were "adapted" for cats by modifying several items. However, no theoretical or psychometric justification is provided for assuming that the underlying constructs remain equivalent after such modifications. Internal consistency (Cronbach's alpha) demonstrates reliability but does not establish construct validity.

I therefore recommend that the authors discuss more explicitly:

* why human infant temperament questionnaires are theoretically appropriate for adult cats,

* how the construct validity of the adapted questionnaires can be justified,

* and what limitations should be acknowledged regarding this adaptation.

---

**2. Why were existing feline personality instruments not used?**

The manuscript itself introduces several feline-specific personality assessments, including the Domestic Cat Personality Inventory, Fe-BARQ, and other established instruments.

However, the manuscript does not adequately explain why these existing feline personality questionnaires were not used, either as primary measures or for convergent validation of the adapted CCTI and IBQ-R.

This omission weakens the methodological justification of the study.

At minimum, the authors should explain why these existing instruments were considered unsuitable for testing their hypothesis, or discuss the limitations of relying exclusively on adapted human questionnaires.

---

**3. Temperament versus personality trait**

Another conceptual issue concerns the developmental stage of the subjects.

IBQ-R was originally developed to assess temperament during infancy, whereas the present study investigates adult cats (1–7 years old). In developmental psychology, temperament and mature personality traits are conceptually distinct constructs.

The manuscript would benefit from a discussion explaining why an infant temperament framework is appropriate for assessing stable behavioral tendencies in adult cats. Without such discussion, readers may question whether the constructs being measured correspond to those originally intended by the questionnaire.

---

**4. Logical progression across Studies 1–3**

The overall structure of the manuscript could be clarified.

Study 1 proposes a clear confirmatory hypothesis based on previous findings in human infants.

Study 2 appropriately attempts to replicate the findings with a larger sample of male cats.

However, the primary confirmatory hypothesis—the predicted quadratic relationship between shyness and approach—was not supported in either Study 1 or Study 2.

After these negative findings, the manuscript shifts its emphasis to hierarchical cluster analysis, an exploratory analysis, and subsequently interprets one cluster as representing "human infant-like shyness."

Although exploratory analyses are valuable for generating new hypotheses, they should be clearly distinguished from confirmatory analyses.

I therefore recommend that the authors explicitly distinguish between hypothesis-testing analyses and hypothesis-generating analyses throughout the manuscript and moderate conclusions derived from exploratory findings.

---

**5. Interpretation of the cluster analysis**

The hierarchical cluster analysis itself is acceptable as an exploratory method.

However, I am concerned that the conclusions may overstate what this analysis demonstrates.

Cluster analysis is an unsupervised exploratory technique. It suggests patterns within the observed data but does not establish the existence of objectively distinct personality phenotypes.

Expressions such as "identified four personality phenotypes" appear stronger than warranted by the evidence. More cautious wording (e.g., "suggested four clusters" or "identified four candidate personality profiles") would better reflect the exploratory nature of the analysis.

Furthermore, the clustering variables include shyness and fear scores that are themselves highly correlated. The authors should discuss the possible influence of correlated variables on the clustering solution.

---

**6. Statistical interpretation**

Overall, the statistical methods selected for the confirmatory hypotheses (Spearman correlations and quadratic regression) are appropriate.

However, the interpretation of the statistical results deserves more careful consideration.

In both Study 1 and Study 2, the quadratic relationship between shyness and approach was not statistically significant. Therefore, the primary hypothesis was not supported.

Subsequent exploratory analyses should not be interpreted as providing equivalent evidence for the original hypothesis.

I recommend that the authors more clearly separate the evidence supporting the original hypothesis from exploratory observations that require future independent replication.

### Minor comments

1. In the Methods section (Study 1), the manuscript uses the first-person singular expression "I changed..." when describing questionnaire modification. This appears inconsistent with the rest of the manuscript and is likely an editorial oversight.

2. The manuscript would benefit from a more explicit discussion of the limitations of owner-reported questionnaires, particularly when assessing internal motivational states such as approach and fear.

3. If feasible, reporting additional effect size measures and discussing the robustness of the cluster solution (e.g., through alternative clustering validation methods) would further strengthen the manuscript.

### Overall assessment

This manuscript presents an interesting and original attempt to extend a developmental psychological framework to comparative animal research. I believe the study has the potential to make a meaningful contribution to the literature.

Nevertheless, the manuscript currently relies on adapted human infant questionnaires without sufficiently establishing their conceptual validity for adult cats, and the distinction between confirmatory and exploratory analyses should be considerably clarified.

Addressing these issues would substantially strengthen both the theoretical contribution and the methodological rigor of the study.

Reviewer #3: Thank you for the opportunity to review this manuscript.

This manuscript investigates whether shyness in domestic cats shares the same multidimensional structure as infant shyness in humans, focusing on the combination of fear and approach tendencies. The study addresses an original and interesting research question by adapting questionnaires developed for human infants to assess cat personality.

However, I have several concerns regarding the overall research framework. While the rationale and hypothesis for Study 1 are clearly presented in the Introduction, the subsequent studies introduce additional research questions that are not sufficiently supported by theoretical or empirical background. Specifically for the sex-specific analyses and the investigation of the effects of human interaction are presented as exploratory analyses, some scientific rationale for conducting these analyses should still be provided. Even for exploratory analyses, a brief theoretical or empirical background would help readers understand why these analyses were performed and how they relate to the overall research question.

Additionally, several conclusions appear to be based primarily on descriptive statistics rather than formal statistical comparisons. For example, statements regarding the distribution of stray-born cats across clusters and the tendency for younger cats to belong to a particular cluster are not accompanied by statistical tests. While descriptive summaries are useful for presenting the data, they are insufficient to support inferential conclusions. If the authors intend to interpret differences among clusters or use these observations to support their hypotheses, appropriate statistical comparisons should be conducted. Otherwise, these findings should be described more cautiously as descriptive observations rather than evidence supporting the proposed interpretations.

The other comments and suggestions for revision are listed below.

Other comments:

Line 24: Please clarify that the numbers reported refer to the number of cats included in Studies 1, 2, and 3, respectively. At this point in the manuscript, it is also difficult for readers to understand why Study 2 focused exclusively on male cats and why Study 3 was further restricted to stray-born male cats. A brief explanation or cross-reference would improve the readability of the manuscript.

Line 32, 35: Please replace "feline" with "cat" throughout the manuscript for consistency.

Line 33: Could you clarify why the results of the cluster analysis support the conclusion that shyness is predominantly associated with fear?

Line 71: Please italicize “Felis catus” throughout the manuscript.

Line 73: Is the term "semi-domestication" commonly used in literature?

Line 75: Please provide the references supporting the statement "earlier research."

Line 80: Please provide the references supporting the statement "earlier studies."

Line 145-146: Can "going shopping" for human infants be considered equivalent to "going to the hospital" for cats?

Line 150, 151: Could you clarify whether the items "My cat makes friends easily" and "My cat is very sociable" refer to interactions with humans or with other cats? The intended target of these questions is unclear, and different respondents may have interpreted them differently. Please clarify the intended context and how respondents were instructed to answer these items.

Line 181-183: Please clarify the rationale for using a regression model with a quadratic term. It would be helpful to explain why a quadratic relationship was hypothesized.

Line 199-207: The authors should be clarifying the rationale for conducting separate analyses for male and female cats. Do the authors hypothesize that different mechanisms underlie shyness in male and female cats? Even if these analyses were exploratory, some theoretical or empirical background should be provided to explain why sex differences were considered relevant and why sex-specific analyses were performed.

Line 208-215: The low statistical power should not be attributed solely to the limited sample size. Given that the observed effect size was also relatively small, an alternative explanation is that the quadratic relationship is genuinely weak or absent in male cats. This possibility should be considered and discussed alongside the explanation of insufficient statistical power.

Line 282: Please clarify the rationale for conducting the cluster analysis. It is currently unclear what specific research question this analysis was intended to address and how it relates to the overall hypothesis of the study.

Line 283: Please clarify how the comparisons among the clusters were conducted. Were the conclusions based solely on descriptive statistics? Given the sample size, it would be more appropriate to statistically compare the clusters (e.g., for age, birth background, or other relevant variables) to support the interpretations presented in the Results and Discussion.

Line 318-319: The statement that the higher proportion of stray-born cats in Cluster 3 "was closer to the hypothesis" is difficult to understand. The manuscript does not appear to present an a priori hypothesis regarding the distribution of stray-born cats across clusters. Please clarify which hypothesis this result supports

Line 386-388: Could you clarify why they hypothesized that this pattern would be observed specifically in male cats? The biological or behavioral rationale for focusing on males is currently unclear. If there is evidence suggesting that the mechanisms underlying shyness differ between male and female cats, this should be described and appropriately referenced.

Line409: Study 2 and Study 3 investigate different hypotheses using different statistical analyses. Therefore, it is unclear why the sample size calculated for Study 2 was directly applied to Study 3. If the primary outcome and statistical approach differed, a study-specific sample size justification would seem more appropriate.

Line 443-446: Statistical comparisons among the clusters should be conducted. Given that the manuscript discusses differences in variables such as age and birth background across clusters, these differences should be formally tested using appropriate statistical methods rather than relying solely on descriptive statistics.

Line 491-492: Could you clarify the basis for the statement that a "potential quadratic relationship" was observed in male cats? As the quadratic term was not statistically significant, it is unclear which results support this interpretation.

Line 505: Despite common perception of uniform behavior in domestic animals; Please provide a reference to support the statement.

**********

what does this mean?). If published, this will include your full peer review and any attached files.

If you choose “no”, your identity will remain anonymous but your review may still be made public.

**Do you want your identity to be public for this peer review?**  For information about this choice, including consent withdrawal, please see our Privacy Policy

Reviewer #1: No

Reviewer #2: No

Reviewer #3: No

**********

[NOTE: If reviewer comments were submitted as an attachment file, they will be attached to this email and accessible via the submission site. Please log into your account, locate the manuscript record, and check for the action link "View Attachments". If this link does not appear, there are no attachment files.]

To ensure your figures meet our technical requirements, please review our figure guidelines: https://journals.plos.org/plosone/s/figures

You may also use PLOS’s free figure tool, NAAS, to help you prepare publication quality figures: https://journals.plos.org/plosone/s/figures#loc-tools-for-figure-preparation.

NAAS will assess whether your figures meet our technical requirements by comparing each figure against our figure specifications.

# Author comment: Author response to Decision Letter 1

**DOI:** 10.1371/journal.pone.0360035.r002

11 Sep 2026

9th September, 2026

Dr. Brittany N. Florkiewicz,

Academic Editor

PLOS ONE

Dear Editor:

On behalf of my co-authors, I wish to re-submit the manuscript titled “Exploration of complex shyness and the relationship between fear and approach behaviors in domestic cats (Felis catus)” The manuscript ID is PONE-D-26-29153.

The manuscript has been rechecked and appropriate changes have been made in accordance with the reviewers’ suggestions. The revised text in our manuscript is visible as tracked changes. The responses to the comments have been prepared and attached herewith. Our responses to comments are in red.

We thank you and the reviewers for your thoughtful suggestions and insights, which have enriched the manuscript and produced a better and more balanced account of the research. We hope that the revised manuscript is now suitable for publication in your journal.

Thank you for your consideration. I look forward to hearing from you.

Sincerely,

Dr. Madoka Hattori

Kyoto University, Kyoto, Japan

E-mail address: hattorimadoka@gmail.com

Additional Editor Comments:

In response to your request regarding the funding disclosure and the role of the funders, we would like to provide the following amended statement:

This study was financially supported by JST SPRING (JPMJSP2110 to M.H.), the Japanese Society of Feline Medicine (JSFM to M.H.), JSPS KAKENHI (24H02200 to S.Y.), and JST FOREST (JPMJFR221I to S.Y.). The funders had no role in study design, data collection and analysis, decision to publish, or preparation of the manuscript.

In accordance with the journal’s instructions, we have also removed the funding-related text from the Acknowledgments section of the revised manuscript. Thank you for your consideration.

Reviewers’ comments:

Response to Reviewers: We sincerely thank the reviewers for their careful evaluation of our manuscript and for their constructive and helpful comments. We have revised the manuscript accordingly and addressed each comment point by point below. All changes made in response to the reviewers’ comments are indicated in the revised manuscript. Line numbers cited in our responses refer to the clean revised manuscript.

Reviewer #1’ comments:

Please consider the following points:-

Response: We thank the reviewer for the careful evaluation of our manuscript and for the constructive comments. We have revised the Introduction to provide a clearer behavioral definition of complex shyness and to explain the theoretical rationale underlying our original hypothesis, as detailed below.

1. Behavioral Definition: What specific, observable feline behaviors would represent this "complex shyness" in a daily setting? For instance, how does a cat with "high fear and high approach" behave differently from a purely "sociable" or purely "fearful" cat?

Response: Thank you for this comment. We have expanded the Introduction to define complex shyness in behavioral terms. For example, when an unfamiliar person enters the cat’s home, a cat with high fear and high approach tendencies may initially retreat but continue to monitor the person from a distance and gradually emerge or move closer. This hypothesized pattern differs from that of a sociable cat, which may approach readily with little initial avoidance, and from that of a fearful cat, which may remain hidden and show little tendency to approach. We have added this behavioral example to the Introduction (Lines 103–108).

2. Theoretical Mechanism: Please elaborate on the psychological or evolutionary mechanism behind the U-shaped quadratic curve. Why would cats with moderate shyness scores decrease their approach behaviors, while cats with extreme shyness scores (very high) still display approach? Providing a clearer theoretical bridging from human infant literature [4] to feline behavioral ecology would significantly strengthen the rationale of this study.

Response: Thank you for this important comment. We have clarified the theoretical basis of the predicted relationship. Based on the human infant literature [4], we hypothesized that fear and approach tendencies may coexist in highly shy individuals. Because neither preverbal infants nor cats can verbally describe this conflict, it must be inferred from observable behavior or caregiver reports. We applied this framework to cats as a working hypothesis. We have revised the Introduction to clarify this theoretical rationale (Lines 101–112).

Reviewer #2’ comments:

Response: We thank the reviewer for the careful and constructive comments. We agree that the manuscript required clearer justification for the adapted questionnaires and a stricter distinction between confirmatory and exploratory analyses. We have revised the manuscript accordingly and moderated the interpretation of the cluster analyses.

Major comments

1. Justification for adapting human infant questionnaires to adult cats

The most important concern is the use of the Colorado Childhood Temperament Inventory (CCTI) and the Infant Behavior Questionnaire-Revised (IBQ-R) for adult domestic cats. The Introduction clearly explains why the authors wished to test the same theoretical framework that has been applied to human infants. However, using the same theoretical framework does not automatically justify using the same measurement instruments. The manuscript states that the questionnaires were "adapted" for cats by modifying several items. However, no theoretical or psychometric justification is provided for assuming that the underlying constructs remain equivalent after such modifications. Internal consistency (Cronbach's alpha) demonstrates reliability but does not establish construct validity. I therefore recommend that the authors discuss more explicitly:

* why human infant temperament questionnaires are theoretically appropriate for adult cats,

* how the construct validity of the adapted questionnaires can be justified,

* and what limitations should be acknowledged regarding this adaptation.

Response: We thank the reviewer for this important comment. We selected the CCTI and IBQ-R because they separately assess shyness, fear, and approach, which were required to test the approach–avoidance hypothesis derived from the human infant literature. Our adaptation was also based on a methodological parallel between the two research settings. In human infant research, behavioral tendencies expressed in relation to familiar caregivers and unfamiliar individuals are assessed by asking an adult caregiver to report the infant’s behavior. In the present study, we applied the same proxy-report structure to the cat–human relationship: an adult owner reported the cat’s observable behavior in relation to its owner, unfamiliar people, and novel situations. Thus, in both settings, an adult who knows the nonverbal subject well evaluates its behavior across everyday social contexts. This parallel provides a rationale for adapting the questionnaires to cats, although it does not imply that the underlying psychological constructs or attachment processes are identical in human infants and adult cats. We have added this methodological rationale to the revised manuscript and have also addressed the remaining issue of construct validity for the adapted CCTI and IBQ-R in the Limitations section (Lines 114-125 and 590-603).

2. Why were existing feline personality instruments not used?

The manuscript itself introduces several feline-specific personality assessments, including the Domestic Cat Personality Inventory, Fe-BARQ, and other established instruments. However, the manuscript does not adequately explain why these existing feline personality questionnaires were not used, either as primary measures or for convergent validation of the adapted CCTI and IBQ-R. This omission weakens the methodological justification of the study. At minimum, the authors should explain why these existing instruments were considered unsuitable for testing their hypothesis, or discuss the limitations of relying exclusively on adapted human questionnaires.

Response: We appreciate the reviewer’s suggestion. Existing feline-specific instruments assess broad personality and behavioral traits but do not separately measure shyness, fear, and approach in the form required for our hypothesis. We therefore used the CCTI and IBQ-R to maintain correspondence with the human infant framework. We have clarified this rationale and acknowledged the lack of convergent validation as a limitation. (Lines 85-98 and 598-600).

3. Temperament versus personality trait

Another conceptual issue concerns the developmental stage of the subjects. IBQ-R was originally developed to assess temperament during infancy, whereas the present study investigates adult cats (1–7 years old). In developmental psychology, temperament and mature personality traits are conceptually distinct constructs. The manuscript would benefit from a discussion explaining why an infant temperament framework is appropriate for assessing stable behavioral tendencies in adult cats. Without such discussion, readers may question whether the constructs being measured correspond to those originally intended by the questionnaire.

Response: Thank you for this important comment. We agree that temperament in human infants and mature personality traits in adult cats should not be regarded as conceptually equivalent. Our intention was not to use the IBQ-R as a general personality measure for adult cats, but to adapt specific IBQ-R items assessing fear and approach tendencies, which were central to our hypothesis concerning complex shyness. We have clarified this rationale in the manuscript and added this issue to the Discussion as a limitation of the cross-species adaptation (Lines 99-125 and 590-614).

4. Logical progression across Studies 1–3

The overall structure of the manuscript could be clarified.

Study 1 proposes a clear confirmatory hypothesis based on previous findings in human infants.

Study 2 appropriately attempts to replicate the findings with a larger sample of male cats.

However, the primary confirmatory hypothesis—the predicted quadratic relationship between shyness and approach—was not supported in either Study 1 or Study 2. After these negative findings, the manuscript shifts its emphasis to hierarchical cluster analysis, an exploratory analysis, and subsequently interprets one cluster as representing "human infant-like shyness." Although exploratory analyses are valuable for generating new hypotheses, they should be clearly distinguished from confirmatory analyses. I therefore recommend that the authors explicitly distinguish between hypothesis-testing analyses and hypothesis-generating analyses throughout the manuscript and moderate conclusions derived from exploratory findings.

Response: We agree that the original manuscript did not sufficiently distinguish confirmatory and exploratory analyses. We have revised the Abstract and General Discussion to clarify that the predicted quadratic relationship was not supported, whereas the subsequent cluster analyses were exploratory and hypothesis-generating. We have also moderated the interpretation of these exploratory findings(Lines 23-38 and 558–566).

5. Interpretation of the cluster analysis

The hierarchical cluster analysis itself is acceptable as an exploratory method. However, I am concerned that the conclusions may overstate what this analysis demonstrates. Cluster analysis is an unsupervised exploratory technique. It suggests patterns within the observed data but does not establish the existence of objectively distinct personality phenotypes. Expressions such as "identified four personality phenotypes" appear stronger than warranted by the evidence. More cautious wording (e.g., "suggested four clusters" or "identified four candidate personality profiles") would better reflect the exploratory nature of the analysis. Furthermore, the clustering variables include shyness and fear scores that are themselves highly correlated. The authors should discuss the possible influence of correlated variables on the clustering solution.

Response: Thank you for this important comment. We agree that hierarchical cluster analysis is exploratory and does not establish objectively distinct personality phenotypes. We have therefore revised the wording to “candidate personality phenotypes” and moderated the interpretation of the clustering results. We have also added a statement to the General Discussion acknowledging that the strong correlation between shyness and fear scores may have influenced the clustering solution (Lines 31–34 and 601–603).

6. Statistical interpretation

Overall, the statistical methods selected for the confirmatory hypotheses (Spearman correlations and quadratic regression) are appropriate. However, the interpretation of the statistical results deserves more careful consideration. In both Study 1 and Study 2, the quadratic relationship between shyness and approach was not statistically significant. Therefore, the primary hypothesis was not supported. Subsequent exploratory analyses should not be interpreted as providing equivalent evidence for the original hypothesis. I recommend that the authors more clearly separate the evidence supporting the original hypothesis from exploratory observations that require future independent replication.

Response: Thank you for this comment. We agree that the exploratory findings should not be interpreted as equivalent evidence for the original hypothesis. As also addressed in response to Comment 4, we have revised the Abstract and General Discussion to explicitly state that the primary confirmatory hypothesis was not supported and to distinguish the subsequent exploratory findings (Lines 29–38 and 549-552).

Minor comments

1. In the Methods section (Study 1), the manuscript uses the first-person singular expression "I changed..." when describing questionnaire modification. This appears inconsistent with the rest of the manuscript and is likely an editorial oversight.

Response: Thank you for pointing this out. We have corrected the first-person singular expression to ensure consistency with the rest of the manuscript (Line 169-171).

2. The manuscript would benefit from a more explicit discussion of the limitations of owner-reported questionnaires, particularly when assessing internal motivational states such as approach and fear.

Response: We agree and have expanded the limitations section to clarify that owner-reported questionnaires reflect interpretations of observable behavior rather than direct measurements of internal motivational states (Lines 586–588).

3. If feasible, reporting additional effect size measures and discussing the robustness of the cluster solution (e.g., through alternative clustering validation methods) would further strengthen the manuscript.

Response: Thank you for this suggestion. In response to this and another reviewer’s comments, we conducted additional comparisons among the clusters (Lines 349–355).

Reviewer #3’ comments:

Response: We thank the reviewer for the careful and constructive assessment. We agree that the rationale connecting Studies 1–3 was not sufficiently explained and that some descriptive observations were interpreted too strongly. We have revised the manuscript accordingly and moderated the interpretation of the cluster analyses.

Line 24: Please clarify that the numbers reported refer to the number of cats included in Studies 1, 2, and 3, respectively. At this point in the manuscript, it is also difficult for readers to understand why Study 2 focused exclusively on male cats and why Study 3 was further restricted to stray-born male cats. A brief explanation or cross-reference would improve the readability of the manuscript.

Response: Thank you for this helpful comment. We have revised Line 24 to explicitly clarify that the three sample sizes correspond to Studies 1, 2, and 3, respectively. We have also briefly explained that Study 2 focused on male cats to follow up on the sex-specific pattern observed in Study 1, whereas Study 3 focused on stray-born male cats to further examine the pattern identified among stray-born cats in Study 2 (Lines 23–29).

Line 32, 35: Please replace "feline" with "cat" throughout the manuscript for consistency.

Response: Thank you for this comment. We have replaced “feline” with “cat” throughout the manuscript for consistency (Lines 39, 612).

Line 33: Could you clarify why the results of the cluster analysis support the conclusion that shyness is predominantly associated with fear?

Response: Thank you for this important comment. We agree that the cluster analysis itself does not demonstrate that shyness is predominantly associated with fear. Rather, the close association between shyness and fear is supported by the strong positive correlations observed between these scores, whereas the exploratory cluster analyses identified a subset of cats with both high fear and high approach scores. We have revised the Abstract to distinguish these two findings more clearly by specifying the analysis supporting each conclusion (Lines 34–38).

Line 71: Please italicize “Felis catus” throughout the manuscript.

Response: Thank you for this comment. We have italicized the scientific name “Felis catus” throughout the manuscript for consistency (Lines 75).

Line 73: Is the term "semi-domestication" commonly used in literature?

Response: Thank you for this comment. The term “semi-domesticated” has been used in the literature to describe domestic cats, reflecting the relatively limited human control over their breeding compared with that of many other domesticated species. However, we agree that the original wording could imply that “semi-domestication” represents a universally accepted classification. We have therefore revised the sentence to clarify that cats have been described as “semi-domesticated” in the literature (Lines 76–82).

Line 75: Please provide the references supporting the statement "earlier research."

Response: Thank you for this comment. We have added references (Bradshaw, 2016) supporting the statement regarding differences in the domestication histories and social behavior of cats and dogs. We have also revised the sentence to remove the statement regarding wider variation in cat personality traits, for which direct comparative evidence is limited, and to more accurately reflect the findings of the cited literature (Lines 79–82).

Line 80: Please provide the references supporting the statement "earlier studies."

Response: Thank you for this comment. We have revised this section to clarify that previous studies of cat personality have examined a variety of personality traits other than shyness, and have explicitly cited examples of these previous studies [18,19] (Lines 85–98).

Line 145-146: Can "going shopping" for human infants be considered equivalent to "going to the hospital" for cats?

Response: Thank you for pointing this out. The statement that “going shopping” was changed to “going to the hospital” did not accurately describe the item used in the actual survey. During manuscript preparation, provisional wording was used because the complete questionnaire items could not be reproduced owing to copyright restrictions, and this wording was inadvertently retained in the submitted manuscript. We have therefore corrected the text to describe an item adaptation that was actually used in the survey. Specifically, “Quickly becomes friendly with other children” was modified to “Quickly becomes friendly with familiar people (non-family members with whom the cat has interacted several times).” This adaptation retained the original item’s focus on becoming friendly with familiar social partners. The manuscript has been revised accordingly (Lines 169–171).

Line 150, 151: Could you clarify whether the items "My cat makes friends easily" and "My cat is very sociable" refer to interactions with humans or with other cats? The intended target of these questions is unclear, and different respondents may have interpreted them differently. Please clarify the intended context and how respondents were instructed to answer these items.

Response: Thank you for this comment. All items in this questionnaire referred to the cats’ behavior toward humans. At the beginning of the questionnaire, respondents were explicitly instructed to answer the questions based on how their cats behaved toward humans. Therefore, “My cat makes friends easily” and “My cat is very sociable” were intended to refer to interactions with humans rather than with other cats. To preserve the wording of the items actually administered to respondents, we did not modify the individual items. Instead, we added the introductory instruction to the Methods to clarify the context in which respondents answered these items(Lines 172–178).

Line 181-183: Please clarify the rationale for using a regression model with a quadratic term. It would be helpful to explain why a quadratic relationship was hypothesized.

Response: Thank you for this comment. The quadratic term was included to test our hypothesis that the relationship between shyness and approach scores would be nonlinear, with the association differing across low, intermediate, and high levels of shyness rather than following a simple linear pattern. This hypothesis was based on the proposed coexistence of fear and approach tendencies at high levels of shyness. We have clarified this hypothesis and its theoretical rationale in the Introduction, as well as the rationale for including the quadratic term in the Statistical Analysis section(Lines 101-112 and 208–211).

Line 199-207: The authors should be clarifying the rationale for conducting separate analyses for male and female cats. Do the authors hypothesize that different mechanisms underlie shyness in male and female cats? Even if these analyses were exploratory, some theoretical or empirical background should be provided to explain why sex differences were considered relevant and why sex-specific analyses were performed.

Response: Thank you for this helpful comment. We did not have an a priori hypothesis that different mechanisms underlie shyness in male and female cats. However, previous research has reported sex differences in several behavioral traits in cats, including nervousness (Takeuchi & Mori, 2009). We have added this background and clarified that the sex-specific analyses were exploratory(Lines 216–219).

Line 208-215: The low statistical power should not be attributed solely to the limited sample size. Given that the observed effect size was also relatively small, an alternative explanation is that the quadratic relationship is genuinely weak or absent in male cats. This possibility should be considered and discussed alongside the explanation of insufficient statistical power.

Response: Thank you for this important comment. We agree that the nonsignificant result in male cats should not be attributed solely to the limited sample size, because the quadratic relationship may in fact be weak or absent. However, the possibility that the relationship was not detected because of the small number of male cats in Study 1 could not be excluded. We have revised the Discussion to clarify that Study 2 was conducted with an expanded sample of male cats to examine these alternative explanations (Lines 250–254).

Line 282: Please clarify the rationale for conducting the cluster analysis. It is currently unclear what specific research question this analysis was intended to address and how it relates to the overall hypothesis of the study.

Response: Thank you for this comment. We have clarified the rationale for the exploratory cluster analysis. Specifically, the analysis was conducted to examine whether distinct groups characterized by different combinations of shyness, fear, and approach could be identified, particularly a subgroup showing both high fear and high approach tendencies as predicted by the complex shyness hypothesis. We have added this rationale to the Statistical analysis section (Lines 313–320).

Line 283: Please clarify how the comparisons among the clusters were conducted. Were the conclusions based solely on descriptive statistics? Given the sample size, it would be more appropriate to statistically compare the clusters (e.g., for age, birth background, or other relevant variables) to support the interpretations presented in the Results and Discussion.

Response: We thank the reviewer for this helpful suggestion. In the original manuscript, the comparisons of age and childhood background among clusters were based on descriptive statistics. Following the reviewer’s suggestion, we conducted additional post hoc comparisons among the four clusters. Cat age did not significantly differ among clusters (Kruskal–Wallis test, χ²(3) = 1.54, p = .673), and childhood background was not significantly associated with cluster membership (Fisher’s exact test, p = .201). As these analyses were not included in our preregistration, we have explicitly identified them as post hoc analyses in the revised manuscript(Lines 349-350, 354–355 and 497-502).

Line 318-319: The statement that the higher proportion of stray-born cats in Cluster 3 "was closer to the hypothesis" is difficult to understand. The manuscript does not appear to present an a priori hypothesis regarding the distribution of stray-born cats across clusters. Please clarify which hypothesis this result supports

Response: Thank you for this comment. We agree that there was no a priori hypothesis regarding the distribution of stray-born cats across clusters. We have therefore removed the statement that this result “was closer to the hypothesis” and revised the text accordingly (Line 356).

Line 386-388: Could you clarify why they hypothesized that this pattern would be observed specifically in male cats? The biological or behavioral rationale for focusing on males is currently unclear. If there is evidence suggesting that the mechanisms underlying shyness differ between male and female cats, this should be described and appropriately referenced.

Response: Thank you for this comment. We did not have an a priori hypothesis that the mechanisms underlying shyness differ between male and female cats. Study 2 focused on male cats because the exploratory sex-specific analysis in Study 1 showed a positive association between shyness and approach in male cats, but not in female cats. We have revised the text to clarify that the focus on male cats in Study 2 was based on this finding from Study 1 rather than on a prior hypothesis of sex-specific mechanisms. We have also clarified that Study 3 focused on stray-born male cats because Study 2 identified the Human infant-like shyness type among cats with this natal background (Lines 425–432).

Line409: Study 2 and Study 3 investigate different hypotheses using different statistical analyses. Therefore, it is unclear why the sample size calculated for Study 2 was directly applied to Study 3. If the primary outcome and statistical approach differed, a study-specific sample size justification would seem more appropriate.

Response: Thank you for this comment. As preregistered, the target sample size for Study 3 was set at 114, following the sample size used in Study 2. We agree that the sample size calculation for Study 2 does not directly justify the sample size for the different analyses conducted in Study 3. We have therefore clarified the preregistered basis for the sample size and additionally conducted a sensitivity power analysis for the correlation analysis in Study 3. We have therefore clarified that the target sample size followed the preregistered design and was not based on a study-specific power analysis (Lines 455-457).

Line 443-446: Statistical comparisons among the clusters should be conducted. Given that the manuscript discusses differences in variables such as age and birth background across clusters, these differences should be formally tested using appropriate statistical methods rather than relying solely on descriptive statistics.

Response: We thank the reviewer for this helpful suggestion. Following the reviewer’s suggestion, we conducted additional statistical comparisons among the four clusters. Cat age differed significantly among the clusters (Kruskal–Wallis test, χ²(3) = 8.25, p = .041); however, post hoc pairwise comparisons with Holm correction showed no significant differences between individual clusters (all adjusted p > .05). The number of months of breeding did not significantly differ among the clusters (Kruskal–Wallis test, χ²(3) = 7.35, p = .062). These analyses were not specified in the preregistration and are therefore reported as additional exploratory analyses. We have added these analyses to the Methods and Results sections and revised the Discussion accordingly (Lines 475-478, 497–502 and 521-524).

Line 491-492: Could you clarify the basis for the statement that a "potential quadratic relationship" was observed in male cats? As the quadratic term was not statistically significant, it is unclear which results support this interpretation.

Response: Thank you for this comment. We agree that the term “potential quadratic relationship” was not sufficiently supported because the quadratic term was not statistically significant in male cats. We have therefore removed this interpretation and revised the text accordingly (Lines 549–552).

Line 505: Despite common perception of uniform behavior in domestic animals; Please provide a reference to support the statement.

Response: Thank you for this comment. We agree that this statement was not sufficiently supported. We have therefore removed the statement regarding common perceptions of uniform behavior in domestic animals and revised the text accordingly (Lines 563–566).

We thank the reviewers again for their constructive comments and hope that the revised manuscript satisfactorily addresses all concerns raised.

Attachment Submitted filename: Response to Reviewers.docx

# Peer review: Decision Letter 1

**DOI:** 10.1371/journal.pone.0360035.r003

**Authors:** Brittany Florkiewicz

22 Sep 2026

Exploration of complex shyness and the relationship between fear and approach behaviors in domestic cats (Felis catus)

PONE-D-26-29153R1

Dear Dr. Hattori,

We’re pleased to inform you that your manuscript has been judged scientifically suitable for publication and will be formally accepted for publication once it meets all outstanding technical requirements.

Within one week, you’ll receive an e-mail detailing the required amendments. When these have been addressed, you’ll receive a formal acceptance letter and your manuscript will be scheduled for publication.

An invoice will be generated when your article is formally accepted. Please note, if your institution has a publishing partnership with PLOS and your article meets the relevant criteria, all or part of your publication costs will be covered. Please make sure your user information is up-to-date by logging into Editorial Manager at Editorial Manager® and clicking the ‘Update My Information' link at the top of the page. For questions related to billing, please contact billing support.

If your institution or institutions have a press office, please notify them about your upcoming paper to help maximize its impact. If they’ll be preparing press materials, please inform our press team as soon as possible -- no later than 48 hours after receiving the formal acceptance. Your manuscript will remain under strict press embargo until 2 pm Eastern Time on the date of publication. For more information, please contact onepress@plos.org.

Kind regards,

Brittany N. Florkiewicz, Ph.D.

Academic Editor

PLOS One

Additional Editor Comments (optional):

Reviewers' comments:

Reviewer's Responses to Questions

**Comments to the Author**

Reviewer #1: All comments have been addressed

Reviewer #2: All comments have been addressed

**********

2. Is the manuscript technically sound, and do the data support the conclusions?

Reviewer #1: Yes

Reviewer #2: Yes

**********

3. Has the statistical analysis been performed appropriately and rigorously?

Reviewer #1: Yes

Reviewer #2: Yes

**********

4. Have the authors made all data underlying the findings in their manuscript fully available?

The PLOS Data policy

Reviewer #1: Yes

Reviewer #2: Yes

**********

5. Is the manuscript presented in an intelligible fashion and written in standard English?

Reviewer #1: Yes

Reviewer #2: Yes

**********

Reviewer #1: Regarding the rationale for examining the quadratic relationship between approach and shyness scores, the authors explain that they investigated this relationship in cats because a similar pattern has been reported in human infants. While this provides a rationale based on cross-species similarity, it does not provide the mechanistic explanation I had hoped to see. In particular, the response does not sufficiently explain why such a quadratic relationship should emerge from a psychological or behavioral mechanism in cats. Nevertheless, considering that the present study is exploratory in nature, I do not consider this limitation to be a sufficient reason to preclude publication in PLOS ONE. Overall, I am satisfied with the authors' responses and revisions and therefore recommend acceptance of the manuscript.

Reviewer #2: (No Response)

**********

what does this mean?). If published, this will include your full peer review and any attached files.

If you choose “no”, your identity will remain anonymous but your review may still be made public.

**Do you want your identity to be public for this peer review?**  For information about this choice, including consent withdrawal, please see our Privacy Policy

Reviewer #1: No

Reviewer #2: No

**********

# Editor report: Acceptance letter

**DOI:** 10.1371/journal.pone.0360035.r004

**Authors:** Brittany Florkiewicz

PONE-D-26-29153R1

PLOS One

Dear Dr. Hattori,

I'm pleased to inform you that your manuscript has been deemed suitable for publication in PLOS One. Congratulations! Your manuscript is now being handed over to our production team.

At this stage, our production department will prepare your paper for publication. This includes ensuring the following:

* All references, tables, and figures are properly cited

* All relevant supporting information is included in the manuscript submission,

* There are no issues that prevent the paper from being properly typeset

You will receive further instructions from the production team, including instructions on how to review your proof when it is ready. Please keep in mind that we are working through a large volume of accepted articles, so please give us a few days to review your paper and let you know the next and final steps.

Lastly, if your institution or institutions have a press office, please let them know about your upcoming paper now to help maximize its impact. If they'll be preparing press materials, please inform our press team within the next 48 hours. Your manuscript will remain under strict press embargo until 2 pm Eastern Time on the date of publication. For more information, please contact onepress@plos.org.

You will receive an invoice from PLOS for your publication fee after your manuscript has reached the completed accept phase. If you receive an email requesting payment before acceptance or for any other service, this may be a phishing scheme. Learn how to identify phishing emails and protect your accounts at https://explore.plos.org/phishing.

If we can help with anything else, please email us at customercare@plos.org.

Thank you for submitting your work to PLOS One and supporting open access.

Kind regards,

PLOS One Editorial Office Staff

on behalf of

Dr. Brittany N. Florkiewicz

Academic Editor

PLOS One
